# A subset of lung cancer cases shows robust signs of homologous recombination deficiency associated genomic mutational signatures

**DOI:** 10.1038/s41698-021-00199-8

**Published:** 2021-06-18

**Authors:** Miklos Diossy, Zsofia Sztupinszki, Judit Borcsok, Marcin Krzystanek, Viktoria Tisza, Sandor Spisak, Orsolya Rusz, Jozsef Timar, István Csabai, Janos Fillinger, Judit Moldvay, Anders Gorm Pedersen, David Szuts, Zoltan Szallasi

**Affiliations:** 1 Department of Health Technology, Section for Bioinformatics, Technical University of Denmark, DTU, Kgs. Lyngby, Denmark; 2 Danish Cancer Society Research Center, Copenhagen, Denmark; 3 Computational Health Informatics Program, Boston Children’s Hospital, Boston, MA USA; 4 Department of Medical Oncology, Dana-Farber Cancer Institute, Boston, MA USA; 5 2nd Department of Pathology, SE NAP, Brain Metastasis Research Group, Semmelweis University, Budapest, Hungary; 6 Department of Physics of Complex Systems, Eötvös Loránd University, Budapest, Hungary; 7 Department of Pathology, National Korányi Institute of Pulmonology, Budapest, Hungary; 8 Department of Tumor Biology, National Korányi Institute of Pulmonology-Semmelweis University, Budapest, Hungary; 9 Institute of Enzymology, Research Centre for Natural Sciences, Hungarian Academy of Sciences, Budapest, Hungary

**Keywords:** Non-small-cell lung cancer, Non-small-cell lung cancer

## Abstract

PARP inhibitors are approved for the treatment of solid tumor types that frequently harbor alterations in the key homologous recombination (HR) genes, *BRCA1/2*. Other tumor types, such as lung cancer, may also be HR deficient, but the frequency of such cases is less well characterized. Specific DNA aberration profiles (mutational signatures) are induced by homologous recombination deficiency (HRD) and their presence can be used to assess the presence or absence of HR deficiency in a given tumor biopsy even in the absence of an observed alteration of an HR gene. We derived various HRD-associated mutational signatures from whole-genome and whole-exome sequencing data in the lung adenocarcinoma and lung squamous carcinoma cases from TCGA, and in a patient of ours with stage IVA lung cancer with exceptionally good response to platinum-based therapy, and in lung cancer cell lines. We found that a subset of the investigated cases, both with and without biallelic loss of *BRCA1* or *BRCA2*, showed robust signs of HR deficiency. The extreme platinum responder case also showed a robust HRD-associated genomic mutational profile. HRD-associated mutational signatures were also associated with PARP inhibitor sensitivity in lung cancer cell lines. Consequently, lung cancer cases with HRD, as identified by diagnostic mutational signatures, may benefit from PARP inhibitor therapy.

## Introduction

PARP inhibitors are a promising new class of cancer therapeutic agents that are most effective in tumors with compromised homologous recombination (HR)-mediated DNA repair^1^. They are approved for the treatment of breast, ovarian, prostate, and pancreatic cancer, which are the solid tumor types most frequently associated with loss of function mutations in key HR genes such as *BRCA1/2*^2^. Other tumor types with HR deficiency may also benefit from PARP inhibitor therapy. Therefore, it is clinically relevant to determine the frequency of HR deficiency in those tumor types as well, which are rarely associated with germline *BRCA1/2* mutations. NSCLC cases show somatic mutations in the *BRCA1/2* genes in 5–10% of the cases^3^, and they also harbor mutations in various DNA damage checkpoint genes^4,5^. It is not known, however, how often those mutations lead to the inactivation of the HR pathway in lung cancer. This could be assessed by analyzing the next-generation sequencing-based DNA aberrations profiles of NSCLC cases.

The loss of function of the key HR genes *BRCA1* and *BRCA2* is associated with a range of distinct mutational signatures that include (1) A single nucleotide variation based mutational signature “Signature 3” (also known as the “BRCAness” signature or SBS3 in COSMIC signatures v3)^6,7^; (2) a short insertions/deletions based mutational profile, often dominated by deletions with microhomology, a sign of alternative repair mechanisms joining double-strand breaks in the absence of HR^7–9^; (3) large-scale rearrangements such as non-clustered tandem duplications of a given size range (mainly associated with BRCA1 loss of function) or deletions in the range of 1–10 kb (mainly associated with BRCA2 loss of function)^10^. We have recently shown that several of these DNA aberration profiles are directly induced by the loss of *BRCA1* or *BRCA2* function in experimental model systems^9^. The presence and frequency of these mutational events can be used to calculate clinically applicable composite mutational signatures of HR deficiency such as the HRD score^11^ and HRDetect score^12^.

We analyzed all available whole-genome sequencing (WGS) data from the TCGA lung adenocarcinoma (LUAD) and squamous lung cancer (LUSC) cohorts and determined which of the above listed mutational signatures are present in these cases. Based on analyzing whole-genome (*n* = 42 and *n* = 48, respectively) and whole-exome (*n* = 553 and *n* = 489 samples) data, we estimated the frequency of potentially HR deficient lung cancer cases.

## Results

### Loss of function mutations of HR genes in lung cancer

Detailed analyses on the germline and somatic mutations of DNA repair genes were performed. We identified pathogenic mutations for the *BRCA2* gene in three LUSC (TCGA-66-2766, TCGA-21-5782 and TCGA-66-2744) and two LUAD cases (TCGA-78-7143 and TCGA-78-7156). All three *BRCA2* mutant LUSC cases and one of the *BRCA2* mutant LUAD cases (TCGA-78-7143) were coupled with LOH. In the tumor of the LUSC donor TCGA-66-2744, the allele harboring the pathogenic germline *BRCA2* mutation was lost due to LOH, thus the wild-type allele was retained, and consequently, it is probably not a BRCA2 deficient case. We also found a LUSC (TCGA-21-1083) case with monoallelic pathogenic *BRCA1* mutation (Supplementary Figs. 1–5, Supplementary Tables 1 and 2). A LUAD case (TCGA-64-1680) was also identified with a monoallelic germline mutation of RAD51B, which was recently shown to be associated with HR deficiency^13^ when accompanied with an LOH in the tumor. We hypothesized that some of these cases may exhibit robust signs of HR deficiency-induced mutational signatures (summaries of the likely HR-impaired cases are available in Supplementary Tables 3 and 4).

### HR deficiency-associated mutational signatures in lung squamous carcinoma

**Fig. 1:**
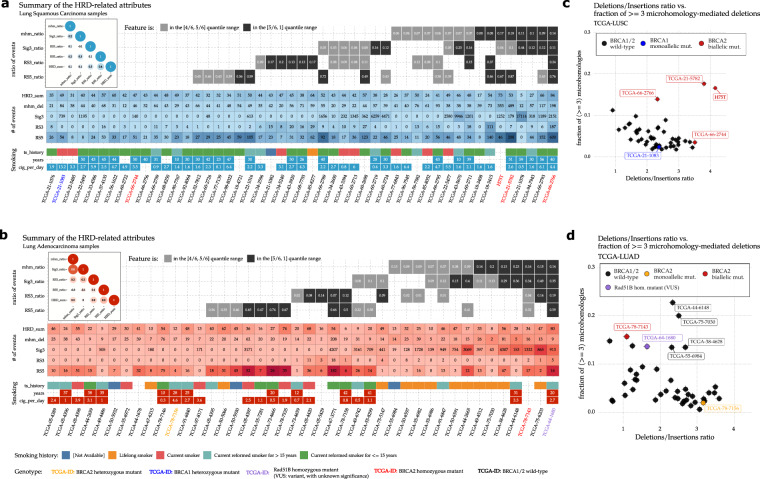
Summary of the HRD-related predictors in the LUAD and LUSC whole-genome dataset. Summary of the HRD-related molecular features extracted from the LUSC (**a**) and LUAD (**b**) whole-genome cohorts. The considered features are as follows: ***HRD_sum*** - the sum of the three allele-specific CNV-derived genomic scars scores (HRD-LOH + LST + ntAI), **RS3_ratio**: Rearrangement signature 3 ratio, i.e., the number of structural variants originating from Rearrangement Signature 3 (RS3), divided by the total number of structural variants in the sample, **RS5_ratio:** Rearrangement Signature 5 ratio, i.e., the number of structural variants originating from rearrangement signatures 5 (RS5), divided by the total number of structural variants in the sample, **mhm_ratio:** the ratio of microhomology-mediated deletions, i.e. the number of deletions, that begin with at least three consecutive nucleotides that it shares with the sequence that follows the deletion (mhm_del), divided by the total number of deletions, **Sig3_ratio:** Substitution signature 3 ratio, i.e., the number of somatic point mutations that can be contributed to the activity of the molecular process captured by Substitution Signature 3 (Sig3), divided by the total number of single nucleotide somatic mutations. The top sections of the two panels contain dark and light gray tiles, which represent the positions of the samples within the distributions of each of the above-described features. A dark gray tile means, that the corresponding ratio (vertical axis) of the sample (horizontal axis) is among the highest within the cohort; it lies between the 5th and 6th sextiles, i.e., above the 83rd percentile. A light gray tile indicates that the corresponding feature ratio is between the 4th and 5th sextiles, i.e., between the 66th and 83rd percentiles. Below the ratio panels, the absolute number of markers is displayed. The bottom section of the panels shows the available tobacco history of the samples. Empty tiles either indicate that at the time of data collection the donor was registered as a non-smoker, or that the information was not available. On the top left sections of the two panels, two correlograms show the Pearson correlation coefficients between all the ratio pairs. Apart from the correlation coefficient between the Signature 3 and microhomology-mediated deletion ratios within the LUAD cohort, all other coefficients were mediocre or negligibly small. Fraction of microhomology-mediated deletions with larger or equal to 3 bp in length, versus the deletions/insertions ratio in the LUSC (**c**) and LUAD (**d**) cohorts.

The two *BRCA2* deficient LUSC cases (pathogenic mutations with LOH, TCGA-66-2766, and TCGA-21-5782) exhibited the highest proportion (>0.1) of at least 3 bp long microhomology-mediated (mhm) deletions (Fig. 1a, b) and the highest proportion of larger than 9 bp long short deletions (Supplementary Fig. 6). The same cases also showed an elevated short deletion/insertion ratio with one of them having the highest such ratio in the cohort (Fig. 1b). Increased deletion/insertion ratios were described previously for *BRCA2*-deficient cancers using WGS data^14^. These two indel patterns have also been described previously in *BRCA2*-deficient human cancer biopsies^8,9,14^.

We did not detect an increased SNV signature 3 (originally described in BRCA1/2 deficient tumors^6^) in these particular cases, probably because the high level of smoking-induced mutational signatures would mask those even if they were present (Fig. 1a) (For a detailed distribution of SNV based signatures see Supplementary Figs. 7–10).

On the other hand, both of the above-mentioned *BRCA2*-deficient cases showed the highest number of RS5 rearrangement signatures (Fig. 1a, Supplementary Fig. 11), which were previously described in breast cancer to be strongly associated with the loss of function of BRCA2^10^.

Taken together, both of the likely *BRCA2*-deficient LUSC specimens showed clear signs of *BRCA2* deficiency associated mutational signatures and thus those cases are likely HR deficient.

### HR deficiency-associated mutational signatures in lung adenocarcinoma

In the case of LUAD, consistent with the lower number of smokers in this tumor type, in about half of the cases (20 out of 42), the smoking signature did not dominate the SNV signatures and the contribution of other mutational processes could be clearly detected (Supplementary Fig. 10). More prominently, the *BRCA2* mutant case with LOH (TCGA-78-7143), along with six other cases, showed a strong presence of signature 3 (Fig. 1c). The same *BRCA2* mutant sample had a high proportion of mhm deletions and four of the other six samples showed high deletion/insertion ratios along with high proportions of mhm deletions (Fig. 1d). The *RAD51B* mutant case (TCGA-64-1680) showed both the signs of the HR deficiency-associated indel patterns and a high signature 3 ratio.

The *BRCA2* mutant case and the four other cases showing HRD-like SNV and indel patterns also showed the presence of the rearrangement signatures associated with *BRCA1/2* deficiency, although to a lesser extent than that seen in TCGA-64-1680 and in *BRCA1/2* mutant breast cancer in general.

### HRDetect scores in the LUAD and LUSC WGS cohorts

Considering that HR deficiency induces different types of mutational signatures, it was proposed that their combination may produce a more accurate measure of HR deficiency than any of those signatures individually. The SNV, short indel and large-scale rearrangement signatures along with a CNV-derived genomic scar score^15^ were combined into a single HRD quantifier, HRDetect^12^. This complex HR deficiency measure was trained on the number and relative distribution of HRD induced DNA aberration profiles in breast cancer.

We calculated the breast cancer trained HRDetect values for all WGS cases by standardizing the lung predictors combined with the original breast cancer dataset, and found that the two above described, likely *BRCA2* deficient LUSC cases; TCGA-66-2766 and TCGA-21-5782 have the highest HRDetect values, the former of which even exceeded 0.7, which was proposed to be the threshold value for bona fide HR deficient cases in breast cancer (Supplementary Fig. 12).

**Fig. 2:**
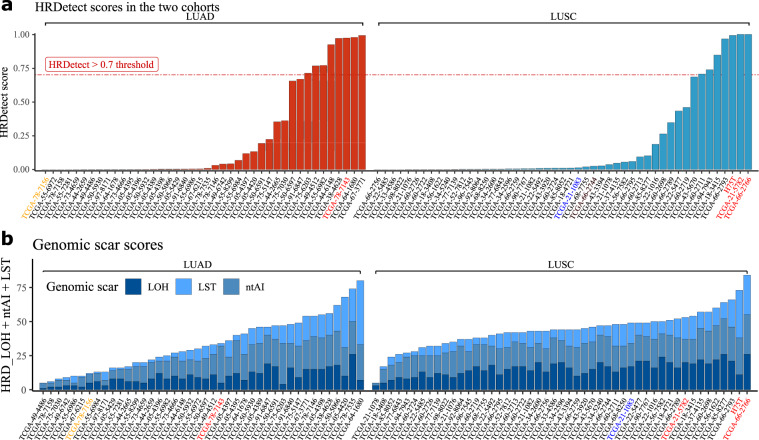
HRD scar scores and HRDetect scores of the LUAD and LUSC WGS datasets. In both panels, the sample names are colored according to their genotypes: yellow—BRCA2 heterozygote mutant, red— BRCA2 homozygote mutant, blue – BRCA1 heterozygote mutant. **a** HRDetect scores calculated using the original, breast cancer whole-genome-based HRDetect weights by following the original article’s standardization and attribute-transformation strategies^12^. **b** The total sum of the genomic scar scores (HRD-LOH, LST, and ntAI) determined from the LUAD and LUSC whole cancer genome’s allele-specific copy-number profiles.

We also calculated HRDetect values standardized on the lung cancer cases alone (Fig. 2a). These values are in general higher since, as we pointed out, some of the HRD suspect cases showed strong signs of some (e.g., SNV and short indel based) HRD signatures but not others (large rearrangement based signatures) (Fig. 1a, c). In other words, it is possible that the individual parameters in a lung cancer-specific HRDetect model will be significantly different from those in breast cancer. Both in the case of LUAD and LUSC, eight of the analyzed cases showed a > 0.7 lung cancer normalized HRDetect value (Fig. 2a) estimating the number of HR deficient cases at less than 20%.

### HR deficiency-associated biomarkers in whole-exome sequencing (WES) data

WES data contain only a fraction, about 1%, of the mutational events present in WGS data. Despite this data sparsity, we showed that in the case of breast cancer WES data can also be used to characterize HR deficiency in tumor biopsies, albeit with lower accuracy^16^. We wanted to determine how well WES data capture HR-deficiency induced mutational signatures in lung cancer (Supplementary Figs. 13 and 14).

We started with the comparative analysis of those cases that had both WGS and WES data available. (Supplementary Fig. 15). For the LUSC cases, the HRD-LOH score showed strong (0.83), and the ratio of signature 3 showed weaker but significant (0.41) correlation across the WES and WGS data. Due to the lower number of detectable deletions in WES data in general, all mhm deletions were considered in WES if their size exceeded 1 bp, and this number was compared to the >2 bp microhomologies in whole genomes. While on average there was a two order of magnitudes difference in the absolute number of deletions between the corresponding pairs, they exhibited a strong correlation (0.79). Of the two most likely HR deficient LUSC cases, TCGA-21-5782 showed good correlation of all three measures across the WES and WGS data. In the other case (TCGA-66-2766), however, the WES data did not recapture the same mutational signatures as the WGS data (Supplementary Fig. 15).

For LUAD all three measures showed correlations between 0.55 and 0.84 across the WGS and WES data, and the cases with high HR deficiency-associated attributes, like signature 3 or the HR deficiency-like insertion/deletion pattern in the WGS data had shown the same tendency in WES data as well.

### HRDetect scores in the LUAD and LUSC WES data

We calculated the HRDetect values based on the standardized (using only the LUAD and LUSC whole exomes) and log-transformed attributes of the WES data (further details of the whole-exome model are available in the Methods). We found only moderate correlations (~0.5) between the corresponding pairs in the lung cancer cohorts (Supplementary Fig. 15). This limited correlation is the likely reason for the lack of enrichment of *BRCA1/2-*deficient cases in the WES cohorts (Supplementary Fig. 16).

We estimated the number of high HRDetect values in the larger, WES cohorts as well and those numbers were lower than those detected in the WGS data. (The proportion was about 16% for LUSC and 4% for LUAD)

Since high HRDetect scores were reported to be associated with better clinical outcome in platinum-treated breast cancer^17^, we were wondering whether lung cancer cases below and above the HRDetect thresholds that we determined in the WES data have significantly different outcome when treated with platinum-containing therapy. However, higher HRDetect scores were not associated with better outcome in these cohorts. (Supplementary Figs. 17 and 18).

### HR deficiency-associated mutational signatures in a lung squamous cell carcinoma case with exceptionally good response to platinum treatment

In order to validate the clinical utility of HRD-associated mutational signatures in lung cancer, we searched our clinical database for advanced lung cancer cases with exceptionally good response to platinum-based therapy and available fresh frozen material for WGS. We identified a stage IVA lung squamous carcinoma case (see case description in the “Methods”) with a durable (>20 months), symptom-free survival in response to platinum-based treatment (H75T). Since the patient was a heavy smoker, the SNV based mutational signatures were dominated by the smoking-associated SNV signature 4 (Supplementary Fig. 10). On the other hand, this tumor showed all short indel consequences of HRD: A high deletion/insertion ratio, high fraction of mhm deletions and high fraction of larger than 10 bp deletions (Fig. 1a, b and Supplementary Fig. 6). It also had a high number of BRCA2 deletion-associated large-scale rearrangement events (1–10 kb deletions) (Supplementary Fig. 11). Finally, the combined HRD measures, the HRD-LOH and HRDetect scores, ranked amongst the highest we analyzed from TCGA (Fig. 2a, b). Finally, the germline DNA analysis uncovered a loss of function *BRCA2* germline variant (rs80358893), which is different from the previously described *BRCA2* germline variants associated with lung cancer^18^. This loss of function mutation in combination with a somatic LOH, likely induced the HRD-associated mutational profiles (Supplementary Figs. 2–4).

### HRDetect scores of lung cancer cell lines are correlated with PARP inhibitor sensitivity

We calculated the HRDetect scores for 67 lung cancer cell lines for which both sequencing data and drug sensitivity data were available in the CCLE and GDSC databases (see “Methods”). Most (*n* = 58, 87%) cell lines had a low HRDetect value <0.25). Eight cell lines had a >0.70 HRDetect score and these were significantly more sensitive to olaparib and talazoparib treatment (Supplementary Fig. 19). They also showed a tendency toward increased sensitivity to cisplatin treatment (Supplementary Fig. 20).

## Discussion

PARP inhibitors show significant clinical efficacy in tumor types that are often associated with *BRCA1/2* mutations, such as breast, ovarian, prostate, and pancreatic cancer. In order to further expand this clinical benefit, there are several ongoing clinical trials evaluating the efficacy of PARP inhibitors in non-small cell lung cancer, such as the PIPSeN (NCT02679963), Lung-MAP (NCT03377556), LODESTAR (NCT04171700), and Jasper (NCT03308942) trials. If, however, the clinical benefit is strongly associated with HRD in this tumor type and only a minority of lung cancer cases harbor this DNA repair pathway aberration, then the success of those clinical trials will greatly depend on our ability to identify and prioritize the HRD cases.

In order to develop such a diagnostic method, we first analyzed the *BRCA1/2* mutant lung cancer cases. Lung cancer is usually not associated with germline *BRCA1/2* mutations, although a few sporadic cases have been reported^18,19^. Nevertheless, due to their often-observed high mutational burden (e.g., due to smoking), about 5–10% of non-small cell lung cancer cases show somatic mutations in either the *BRCA1* or *BRCA2* gene. Some of those are likely to be pathogenic and associated with LOH as well. In our analysis, these cases, when analyzed by WGS, clearly showed the mutational signatures usually associated with HRD in clinical samples^8^ and induced by *BRCA1/2* deficiency in experimental model systems^9^. This strongly implies that there are *bona fide* HRD cases amongst lung cancer as well. Beyond mutations in *BRCA1/2*, HRD can be induced by a variety of mechanisms, such as suppression of expression of *BRCA1* by promoter methylation. However, we did not find any association between the promoter methylation of HR genes and increased HRD-associated mutational signatures (Supplementary Tables 3 and 4, Supplementary Fig. 5).

*BRCA1/2* deficiency and HRD-induced mutational signatures do not show a perfect correlation. A significant number of ovarian and breast cancer cases show clear patterns of HRD-associated mutational signatures in the absence of mutations of *BRCA1/2* or other key HR genes^12^. Conversely, *BRCA1* mutant cases can be rendered HR proficient and PARP inhibitor resistant by the loss of other genes such as *53BP1* or *REV7*^20,21^. Therefore, downstream mutational signatures, such as those investigated in our analysis, could be more accurate measures of HRD than the mutational status or expression change of individual genes and they could serve as a complementary biomarker to the mutation status of HR-associated genes. In fact, we identified several lung cancer WGS cases with high HRD-induced mutational signatures that were not associated with *BRCA1/2* mutations and those could also be responding to PARP inhibitor therapy as our cell line-based preclinical analysis suggests. We made every effort to detect a likely explanation for the cases with significant HRD-associated mutational signatures but TCGA profiles have significant limitations due to e.g. normal tissue contamination. For example, significant expression deficiency or LOH of the *BRCA1* or *BRCA2* genes can often be masked by the presence of these genes in the normal cells in the tumor biopsy.

It is important to estimate the proportion of potentially HR-deficient non-small lung cancer cases to optimize PARP inhibitor trials. Using the limited number (less than one hundred in total), WGS covered lung cancer cases we estimated the proportion of truly HR deficient cases as less than 20%. In our analysis, WES data seemed to be less accurate to determine HRD status in lung cancer. This could be due to, e.g. the high level of smoking-induced mutational signatures interfering with the HRD induced mutational signatures.

We used the WES-derived HRD measures to investigate the correlation between the likely presence of HRD and better survival upon treatment in lung cancer in TCGA. We did not see any correlation, which is probably due to a number of factors. These patients were treated in addition to platinum with other agents as well. Sensitivity or resistance to platinum treatment is also associated with several other mechanisms in addition to HRD^22,23^ and the WES-derived HRD-associated mutational signatures are less accurate than their WGS-based counterparts. Furthermore, response to platinum-based therapy did not translate into improved survival in a large study investigating the association of DNA repair gene polymorphisms with response to platinum-based doublet chemotherapy in non-small-cell lung cancer^24^.

On the other hand, when we investigated the mutational signatures from WGS data of an exceptionally good responder of lung cancer to platinum-based therapy, the high levels of HRD-associated mutational signatures were apparent. This strongly suggests that *bona fide* HR-deficient cases with clinical consequence can be identified, warranting further clinical validation.

## Methods

The matched normal-tumor BAM files of the WES samples were downloaded from the GDC data portal (portal.gdc.cancer.gov). There were *n* = 489 LUSC and *n* = 553 LUAD samples available with both normal and tumor samples. The Mutect2 vcf files and the clinical data were also downloaded from the data portal.

The BAM files for the whole-genome-sequenced samples were downloaded from the ICGC data portal (dcc.icgc.org). (LUSC: *N* = 48, LUAD: N = 42 patients.) The majority of the donors with available whole-genome data, had whole-exome sequences as well. All the 48 patients in the LUSC WGS cohort had at least 1 corresponding whole exome, but out of the 42 LUAD WGS patients only 39 had matching whole exomes. The WGS samples without pairs: TCGA-05-5429, TCGA-64-1678, and TCGA-78-7143. Since TCGA-78-7143 had a likely pathogenic germline BRCA2 mutation coupled with an LOH, for this patient an exonic bam-slice was created using the reads that covered the exome from the WGS bam, in order to check whether the BRCAness phenotype was detectable in the exonic version.

### Mutation, copy number, and structural variant calling

Germline single nucleotide mutations were specifically called at and around the key HR-related genes for genotyping purposes using HaplotypeCaller (GATK v3.8)^25^ while somatic point mutations and indels were called using MuTect2 (GATK v3.8). In order to ensure the high fidelity of the reported SNVs, additional hard filters were applied to the set of identified variants. For germline variants, the minimum mapping quality (PHRED) was set to 50, variant quality to 20, and a minimum coverage of 15 was ensured, while for somatic variants the minimum tumor LOD (logarithm of odds) was set to 6, the minimum normal LOD to 4, the minimum normal depth to 15, the minimum tumor depth to 20, and the minimally allowed tumor allele frequency to 0.05.

Copy-number profiles were called using Sequenza^26^, with fitted models in the ploidy range of [1,5] and cellularity range [0.1,1]. When a fitted model’s predictions significantly differed from the expected ploidy-cellularity values, an alternative solution was selected manually.

Structural variants were detected via BRASS (v6.0.0 - https://github.com/cancerit/BRASS). Through additional hard filters, the minimum amount of variant-supporting read-pairs was set to 6, and a successful local de novo assembly of the reads by velvet was demanded.

### Genotyping

The genotypes of the key HR-related genes were determined via annotating the small-scale variant files (GATK v3.8 HaplotypeCaller) using InterVar^27^. From the resulting variant files only the exonic and the ±10 nucleotide regions around the exons (in order to account for the possible splice-variants) were considered. From these mutations only those were retained, that were predicted as “Likely Pathogenic”, “Pathogenic” or “Uncertain” according to ClinVar (20170905). At last, a threshold on the depth of these variants was set to 20. In case a variant was characterized as pathogenic or likely pathogenic by InterVar, the corresponding sample was considered mutant, assuming that at least a heterozygous mutation was present in the sample. Variants with unknown significance were also collected, but they did not affect the genotyping scheme.

Mutations found in the LUAD WGS cohort are summarized in Supplementary Fig. 1, mutations found in the LUSC WGS cohort are summarized in Supplementary Fig. 2.

The occurrence of the loss of heterozygosity in these genes was estimated using the samples’ sequenza-derived copy-number segments. If the copy-numbers of either the A or B alleles dropped to zero within the coordinates of a gene, then the LOH event was registered (Supplementary Fig. 3).

Methylation probe data (Human Methylation 450k) were downloaded from the ICGC data portal and analyzed by following the Broad Firehose pipeline: http://gdac.broadinstitute.org/runs/stddata__2016_01_28/ (Broad Institute TCGA Genome Data Analysis Center, 2016). Supplementary Tables 3 and 4 contain the tissue-specific probes that were most negatively correlating with the expression values of their corresponding genes. The Pearson correlation coefficients and their corresponding *P* and *Q* values are also shown in the tables. Since the majority of the samples had only Human Methylation 27k data available or did not have methylation info at all (Supplementary Fig. 4), the genotyping scheme did not consider the methylation status of the gene-specific probes.

The final genotypes are illustrated in Supplementary Fig. 5. Likely pathogenic hetero- or homozygous BRCA1/2 mutations were detected in both cohorts. These variants were the following:

**LUAD**:

• TCGA-75-7156 (likely pathogenic BRCA2 germline mutation)

/frameshift insertion at chr13:32912949,T>TTGTGC/

• TCGA-78-7143 (likely pathogenic BRCA2 germline mutation + LOH)

/frameshift insertion at chr13:32906473, A>ACCTAATCTTACTATAT/

**LUSC:**

• TCGA-21-1083 (likely pathogenic BRCA1 somatic mutation)

/stopgain SNV at chr17:41244585, G>C/

• TCGA-21-5782 (likely pathogenic BRCA2 somatic mutation + LOH)

/frameshift deletion at chr13:32930627, AG>A/

• TCGA-66-2744 (likely pathogenic BRCA2 germline mutation + LOH)

/frameshift deletion at chr13:32912337, CTG>C/

• TCGA-66-2766 (likely pathogenic BRCA2 germline mutation + LOH)

/stopgain SNV at chr13:32914349, G > T/

In addition, TCGA-64-1680—a LUAD sample with high HRD-related genomic aberration scores—had a UNK germline mutation in RAD51B /nonsynonymous SNV at chr14:68352672, A>G/.

### Case report of the platinum-sensitive LUSC patient: H75T

A 76-year-old man was diagnosed with solitary pulmonary nodule in the right upper lobe during a screening chest X-ray in August 2016. He was an ex-smoker with 35 pack-year index, and had quitted smoking 20 years ago. He suffered from atherosclerosis and cardiovascular disease. In January 2017, segmental surgical resection of the right upper lobe was performed with a pathological diagnosis of poorly differentiated squamous cell lung carcinoma of 31 mm in diameter with lymphoid vessel invasion (pT2a-N0-M0). He received no adjuvant oncotherapy. In June, 2017 PET-CT revealed bilateral pulmonary dissemination (of maximum 8 × 16 mm) and enlargement of the mediastinal lymph nodes with (18)F-FDG avidity, therefore, gemcitabine-carboplatin chemotherapy was indicated by a multidisciplinary tumor board. After 4 cycles of this treatment partial response could be observed using the RECICST 1.1 criteria. In March, 2018 no evidence of tumor could be demonstrated on chest CT. In April 2019 the patient is still alive.

The study was directed in accordance with the guidelines of the Helsinki Declaration of the World Medical Association. The national level ethics committee (Hungarian Scientific and Research Ethics Committee of the Medical Research Council, No 2285-1/2019/EUIG and 2307-3/2020/EUIG) approved the study.

Written informed consent was obtained from the patient to perform genomic analyses of the tumor and peripheral blood samples. Permissions to use the archived tissue have been obtained from the Regional Ethical Committee (No: 510/2013, 86/2015).

### HRD-related biomarkers

The HRD-induced genomic fingerprints analyzed in this study were the following:

1. Somatic substitution signatures^6^
2. mhm deletion ratio, and insertion/deletion ratio^9,14^
3. Genomic scar scores^15,28,29^
4. Rearrangement Signatures^10^

### Mutational signatures

Somatic point-mutational signatures were estimated with the deconstructSigs R package^30^ (Supplementary Figs. 7–10). The list of considered mutational processes whose signatures’ linear combination could lead to the final mutational catalogs (a.k.a. mutational spectra) was confined to those, that were reportedly present in LUADs and LUSCs according to the COSMIC database (i.e., in LUAD: Signatures 1, 2, 4, 5, 6, 13, and 17, in LUSC: 1, 2, 4, 5, and 13). Furthermore, since we were primarily interested in their HR-related signature composition, we have added Signature 3 and 8 to the lists. After the evaluation of their signature compositions, the mutational catalogs of the samples were reconstructed, and the cosine of the angle between the 96-dimensional original and reconstructed vectors were measured (cosine similarity). Using this technique, we also checked whether the incorporation of any additional signatures would improve the mean reconstruction similarities significantly, but the improvement was negligible in both WGS cohorts (Supplementary Fig. 8). In general, the final cosine similarities were adequately high, especially between the original and reconstructed squamous carcinoma whole genomes (Supplementary Fig. 9). The resulting composition of mutational signatures per sample is presented in Supplementary Fig. 10.

### Classification of deletions

It was shown recently that cancer cells, that exhibit HR deficiency, have unique characteristics in their indel profiles. Specimens with biallelic BRCA1/2 mutations have significantly more deletions that are longer than 10 bp than BRCA1/2 wild-type tumors, and they also tend to have more deletions than insertions^14^. It was also found that these deletions mostly arise due to the activity of the Microhomology Mediated End Joining (MMEJ) or the Single Strand Annealing (SSA) DNA repair pathways, and thus the relative ratio of mhm deletions among them is significantly higher than in HR-competent cases^9^. Since the HR and MMEJ pathways differentiate at the point when RPA binds to the ssDNA overhangs, a dysfunctional BRCA2 protein involuntarily gives rise to an increased MMEJ/SSA activity. Non-surprisingly, the aforementioned increase in the mhm-deletion ratio is much more obvious in samples with BRCA2^−/−^ mutations than in BRCA1^−/−^ tumors.

In general, deletions were classified into three sets: (1) complete repetitions; when the complete deleted sequence is repeated after the deletion in the reference genome, (2) microhomologies; when only the first *n* nucleotides of the deleted sequence are repeated after the deletion and (3) unique deletions, when the sequence following the deletion has no resemblance to the deleted series of nucleotides. However, since the repetition of the first 1-2 nucleotides could occur by pure chance (with 0.25 and 0.0625 probabilities respectively—assuming that all four nucleotides can occur with the same probability), when investigating the effects of the MMEJ/SSA pathway, it is considered a good practice to work with the *n* ≥ 3 microhomologies only. Supplementary Fig. 6 provides a summary of this analysis.

### Rearrangement signatures

For rearrangement signatures, only those variants were considered that had at least six supporting reads that were successfully de novo-assembled by velvet^31^. The extraction of rearrangement signatures was executed according to the following strategy: first, the reported structural variants were mapped to the alphabet of the 32-dimensional structural variant-affecting mutational alphabet as described in the original publication^10^ and stored into the matrices **M**_**LUSC**_ and **M**_**LUAD**_. Due to the low number of samples in the two WGS cohorts, the extraction of de novo rearrangement signatures was not achievable. Instead, a breast cancer-based, previously described matrix of mutational signatures (**P**) was used^10^. From these matrices, the signature composition (**E**) was estimated by solving the non-negative least squares problem ||P*E – **M**||_2_, subject to *E*_ij_ > 0, for all *i* and *j* (Supplementary Fig. 11).

### Genomic scar scores

The calculation of the genomics scar scores (loss-of-heterozygosity: LOH, large-scale transitions: LST and number of telomeric allelic imbalances: ntAI) was performed using the scarHRD R package^32^. The allele-specific segmentation data of the samples were provided by sequenza.

### HRDetect

Due to the absence of a sufficient number of authentic HR-deficient cases, the derivation of two separate, LUAD and LUSC specific HRDetect models was not achievable. Instead, on the whole genomes, the scores were calculated using the original, breast cancer-derived model, while the whole exomes relied on an alternative, whole exome-based, but also breast cancer-specific model.

For the whole genomes, the model weights were the following:

- intercept = −3.3642
- Signature.8 = 0.09062
- HRD-LOH = 0.6666
- RS5 = 0.8467
- RS3 = 1.1532
- Signature.3 = 1.6114
- mhm.del.ratio = 2.3977

The whole-exome model was trained on the 560 artificially derived (from whole genomes) breast cancer whole exomes^16^, with final weights:

- intercept = −2.6192939
- Signature.17 = 0.067098
- Signature.20 = 0.09409
- Signature.26 = 0.16166
- Signature.6 = 0.310146
- Signature.18 = 0.31205
- mhm.del.ratio = 0.314225
- Signature.8 = 0.61474
- Signature.13 = 0.83017
- Signature.3 = 2.00757
- HRD-LOH = 2.3865

In order to reach to the results of Fig. 2a, the whole-genome features were log-transformed and standardized within their respective cohorts (i.e., *N*_LUSC_ = 49, N_LUAD_ = 42), i.e. the *z*-scores of each of the log-transformed attributes were calculated using the means and standard deviations of the 49 LUSC and 42 LUAD samples respectively. Since the original model was trained on 560 breast cancer whole genomes, the standardization step was executed using the means and standard deviations of the 560 breast cancer whole genomes as well. The distributions of these alternative (referred to as “breast cancer standardized”) HRDetect predictions are available in Supplementary Fig. 12, and both the scores and the predictor attributes of HRDetect are available in Supplementary Table 4 and Supplementary Data 1 and 2.

### Comparison of WES and WGS HRDetect scores

The two LUSC patients showing signs of HR-deficiency based on WGS, also had high HRDetect values based on WES analysis (TCGA-21-5782: 0.80, TCGA-66-2766: 0.66). Since the HR deficiency status of these two cases are supported by WGS data, we used the lower HRDetect value of these two cases as a putative threshold for HR deficiency in the WES characterized LUSC cohort. In the LUSC WES cohort 16% of the patients had higher than 0.66 HRDetect scores, while in the case of the LUAD cohort 3.8% of the patients had at least as high HRDetect score as the RAD51B-mutated sample (TCGA-64-1680) (Supplementary Fig. 15).

### Survival analysis

Higher WES-based HRDetect scores were not associated with better progression-free survival (PFS) or overall survival (OS) in LUAD and LUSC patients in the TCGA dataset. There was also no significant difference between platinum-treated and non-treated patients (Supplementary Figs. 17 and 18).

### HRDetect values in cell lines

The mutational information and copy-number profiles of LUAD and squamous cell carcinoma cell lines were obtained from the CCLE^33^. PARP-inhibitor sensitivity was downloaded from the GDSC project^34^ using the DepMap portal^33^. The mutational signatures were estimated using the deconstructSigs R package. Due to the missing reliable allele-specific copy-number data, a simplified HRDetect model was applied using signatures 3,6,8,13, and 17, and the proportion of mhm deletions. This simplified model was trained on the 560 breast cancer artificial WES data by following the strategy of the original publication^12^. The final weights were the following:

intercept = −1.46692,

unique.del.ratio = 0.029158,

Signature.6 = 0.098565,

Signature.17 = 0.186293,

Signature.8 = 0.315059,

mhm.del.ratio = 0.548391,

Signature.13 = 1.100178,

Signature.3 = 1.920547,where unique.del.ratio is the ratio of deletions with no microhomology content.

### Reporting summary

Further information on research design is available in the Nature Research Reporting Summary linked to this article.

## Supplementary information

Supplementary Information

Supplementary Data 1

Supplementary Data 2

Reporting Summary

## Data Availability

The data generated and analyzed during this study are described in the following data record: 10.6084/m9.figshare.14452854^35^. The results shown here are in part based upon data generated by the TCGA Research Network: https://www.cancer.gov/tcga, and the LUAD and LUSC data are available at ICGC (https://dcc.icgc.org/) and GDC (https://portal.gdc.cancer.gov/) data portals. A comprehensive list of the file names underlying the figures and supplementary materials of the related article, along with direct links to the data in the above sources, is provided in the file ‘Diossy_et_al_2021_underlying_data_list.xlsx’, which is included with the data record. Sample single nucleotide variation analysis of the stage IVA lung squamous carcinoma case with durable, symptom-free survival in response to platinum-based treatment (H75T) has been deposited in the European Variation Archive under accession https://identifiers.org/ebi/bioproject:PRJEB45238^36^.

## Code availability

All analysis was done using standard R (v3.6) codes with the help of the following packages: ggplot2, data.table, deconstructSigs, lsa, ggbeeswarm, RColorBrewer, sequenza, copy number, and cluster. In particular, standard variant files were converted to tab-delimited tables using GATK (v3.8) VariantsToTable and manipulated using the data.table package in R. Figures were created using ggplot2. Every tool mentioned in the Methods section were used with default parameters unless stated otherwise.
