# Supplementary material for: A subset of lung cancer cases shows robust signs of homologous recombination deficiency associated genomic mutational signatures
Source: NPJ Precis Oncol. 2021 Jun 18;5:55. doi: 10.1038/s41698-021-00199-8 (PMC8213828; doi:10.1038/s41698-021-00199-8)
Supplement: Supplementary file 1 — Supplementary Information [file 41698_2021_199_MOESM1_ESM.pdf]

Supplementary Figures and Tables

List of Figures

1 LUAD WGS germline and somatic mutations 3
2 LUSC WGS germline and somatic mutations 4
3 LOH in the WGS cohorts 5
4 Array-based methylation states of HR-related genes 8
5 LUAD and LUSC WGS genotypes 9
6 LUAD and LUSC WGS insertions and deletions 10
7 LUAD and LUSC WGS somatic snvs 11
8 Dynamic Signature Extraction Strategy 11
9 Cosine similarities - WGS 12
10 LUAD and LUSC WGS somatic signatures 12
11 LUAD and LUSC WGS SVs and rearrangement signatures 13
12 LUAD and LUSC WGS HRDetect - breast standardized 14
13 LUAD HRD-related genomic features 17
14 LUSC HRD-related genomic features 18
15 Correlations: WES vs WGS HRD-related scores 19
16 LUAD and LUSC WES HRDetect - breast standardized 20
17 Survival plots - LUAD 21
18 Survival plots - LUSC 22
19 Cell lines - sensitivity to PARP inhibitors 23
20 Cell lines - sensitivity to platinum salts 23

List of Tables

1 LUAD - Table of methylation probes 6
2 LUSC - Table of methylation probes 7
3 Summary of LUSC samples mutations in HR-related genes 15
4 Summary of LUAD samples mutations in HR-related genes 16

Supplementary data not included in this document

Supplementary Data 1 and 2 are available separately, in xlsx format.

- **Supplementary Data 1**: List of the **LUAD** whole genome HRD-related attributes, that were used to calculate the HRDetect scores of the samples. Pound signs in front of attribute names indicate that the absolute number of mutations is considered. Values in the regular HRDetect column was calculated by using the standardized and log-transformed values of these attributes alone, while the breast standardized HRDetect values were calculated after the attributes were standardized combined with the predictors of the 560 breast cancer whole genomes.

- **Supplementary Data 2**: List of the **LUSC** whole genome HRD-related attributes, that were used to calculate the HRDetect scores of the samples. Pound signs in front of attribute names indicate that the absolute number of mutations is considered. Values in the regular HRDetect column was calculated by using the standardized and log-transformed values of these attributes alone, while the breast standardized HRDetect values were calculated after the attributes were standardized combined with the predictors of the 560 breast cancer whole genomes.

Color-code

Some of the supplementary figures and tables use colored sampleIDs to indicate the genotypes of a given sample. The color-code follows the legend of main Figure 1:

- TCGA-ID: BRCA2 heterozygous mutant
- TCGA-ID: BRCA1 heterozygous mutant
- TCGA-ID: BRCA2 homozygous mutant
- TCGA-ID: BRCA1/2 wild type

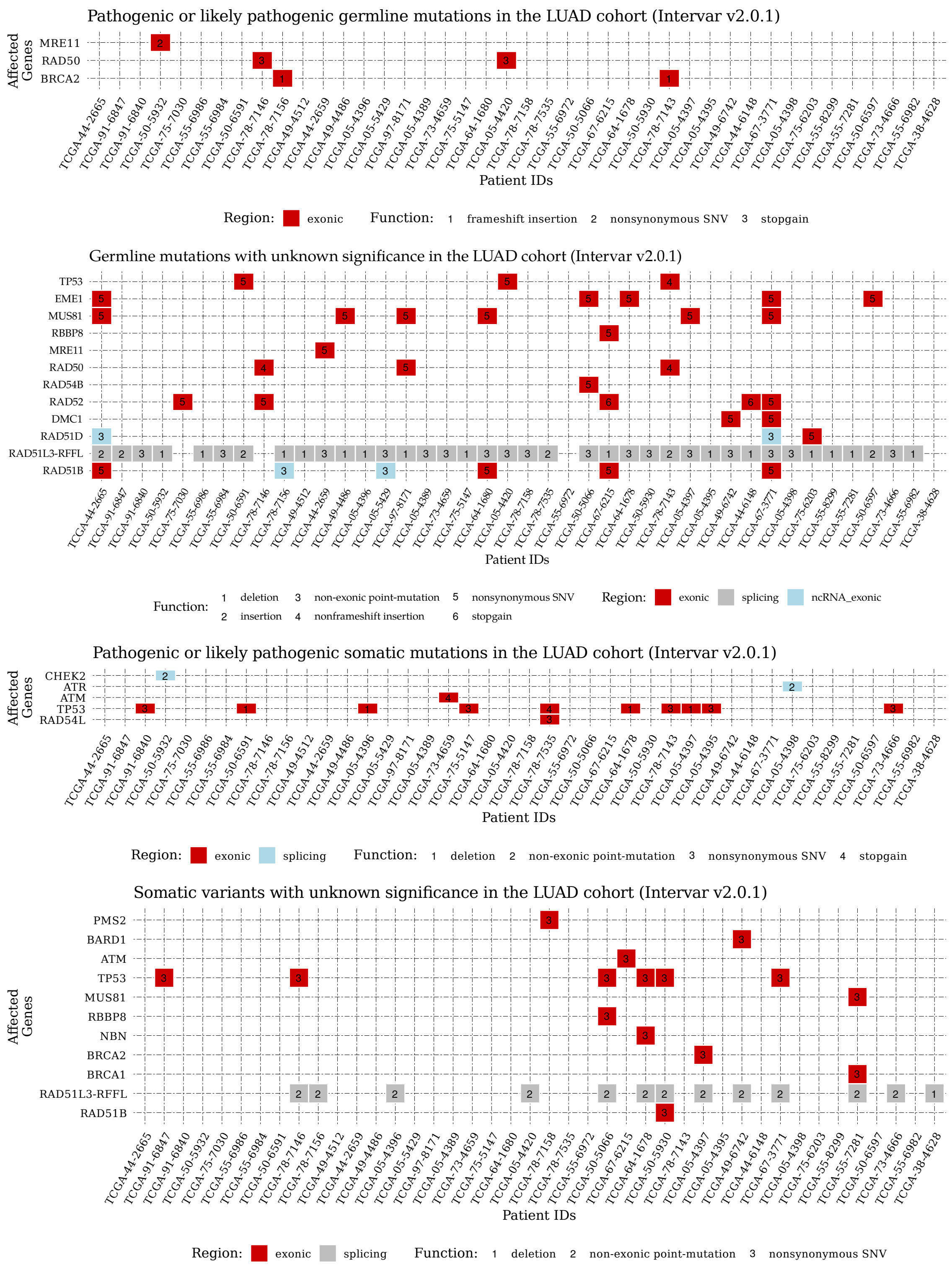

Supplementary Figure 1: *First two panel from the top: Pathogenic or likely pathogenic and UNK germline mutations in the LUAD WGS cohort. Bottom two panel: Somatic variants in the LUAD WGS cohort*

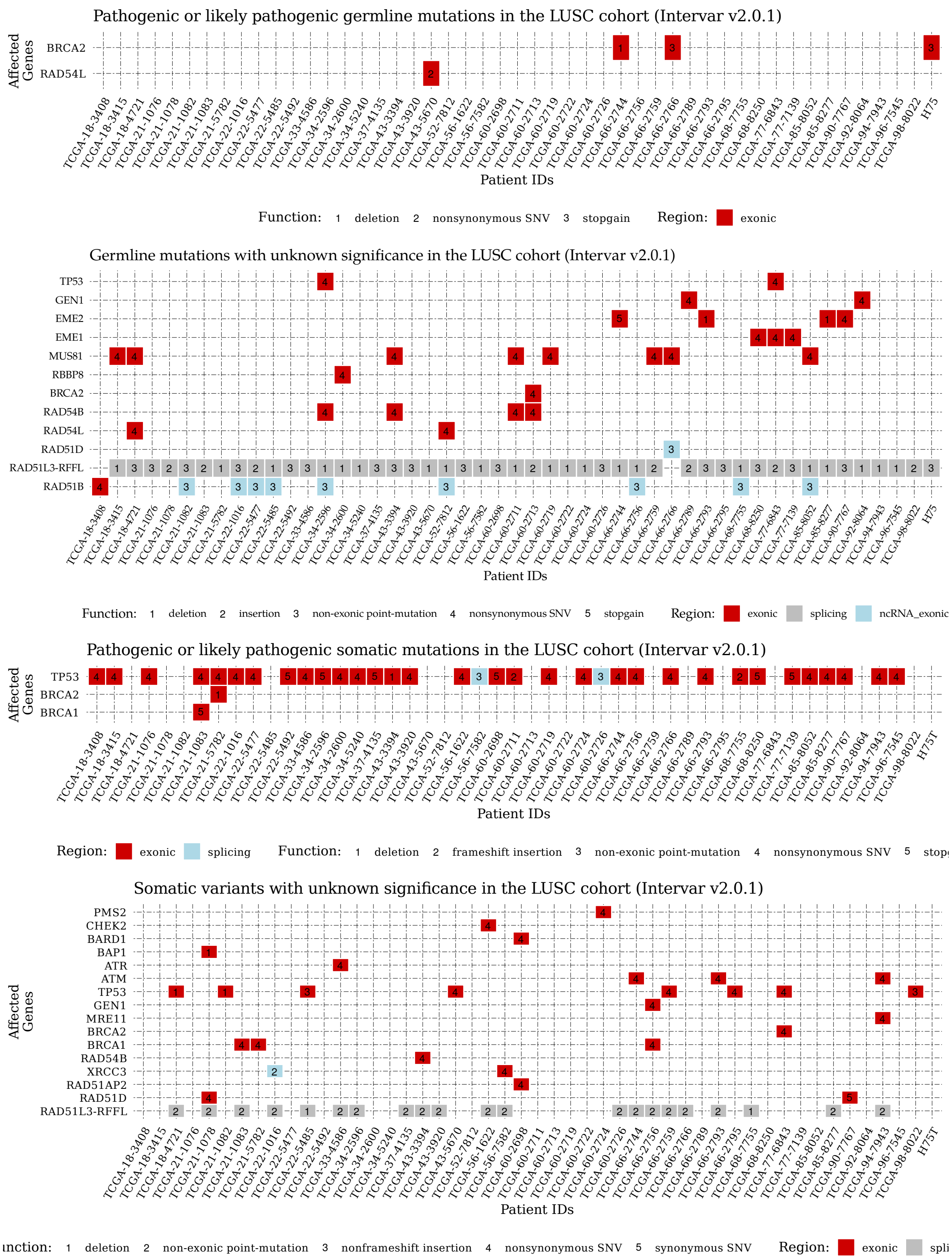

Supplementary Figure 2: *First two panel from the top: Pathogenic or likely pathogenic and UNK germline mutations in the LUSC WGS cohort. Bottom two panel: Somatic variants in the LUSC WGS cohort*

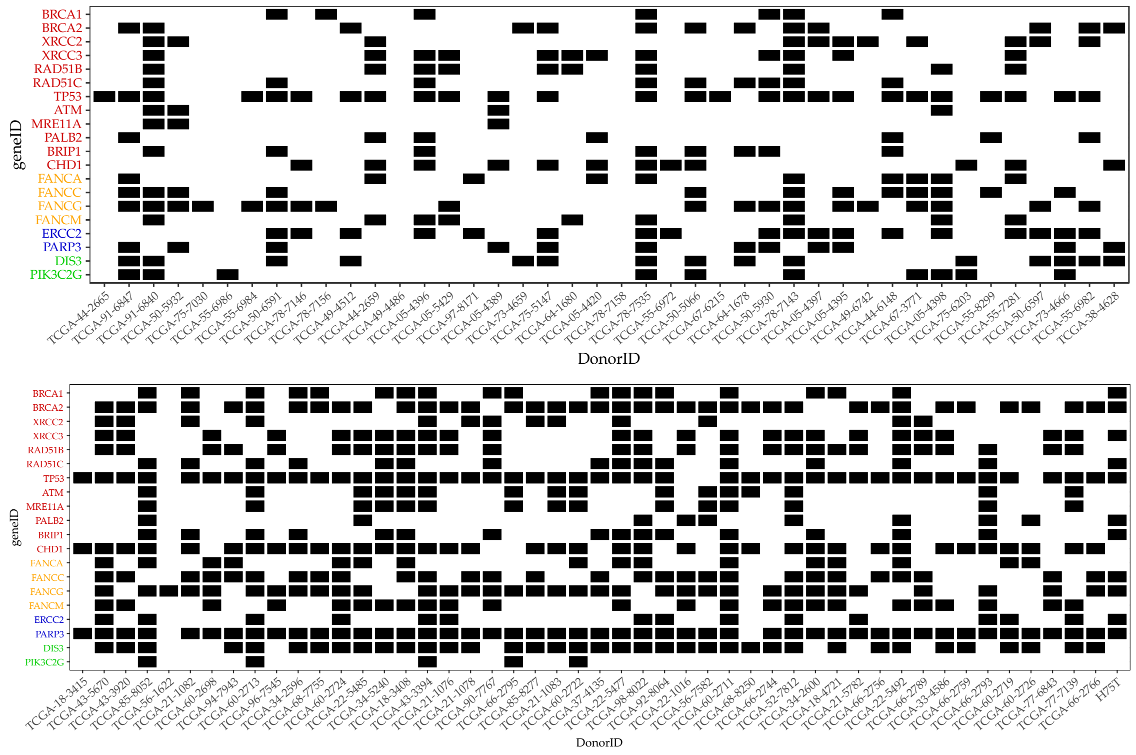

Supplementary Figure 3: *Estimated occurrences of LOH events in the analyzed genes. Segment means were estimated using the sequenza and copynumber R packages.*

Gene	Meth_Probe	Chrom	Position	Corr_Coeff	Pval	Qval	Expr_Mean	Meth_Mean
**PIK3C2G**	cg17881542	12	18415070	-0.494	1.22E-22	4.08E-23	2.474	0.703
**FANCA**	cg07440826	16	89882328	-0.349	2.73E-14	6.92E-15	8.425	0.130
**RAD51C**	cg25339112	17	56769760	-0.307	3.11E-11	6.06E-12	7.904	0.617
**PARP3**	cg27227100	3	51977385	-0.305	3.33E-11	6.45E-12	8.972	0.809
**BRCA2**	cg26458617	13	32889213	-0.255	3.61E-08	5.10E-09	6.742	0.207
**BRCA1**	cg16029534	17	41197783	-0.242	1.95E-07	2.53E-08	8.103	0.875
**FANCG**	cg05293216	9	35080812	-0.229	9.03E-07	1.08E-07	8.211	0.718
**XRCC2**	cg01605516	7	152369723	-0.213	5.07E-06	5.48E-07	5.856	0.870
**ERCC2**	cg27517897	19	45874080	-0.199	2.07E-05	2.04E-06	8.792	0.192
**FANCC**	cg21891967	9	98075492	-0.169	3.05E-04	2.55E-05	7.737	0.472
**PALB2**	cg08762306	16	23652796	-0.164	4.56E-04	3.73E-05	8.321	0.327
**DIS3**	cg14756530	13	73355442	-0.161	5.63E-04	4.55E-05	8.464	0.061
**ATM**	cg14761454	11	108092087	-0.160	6.89E-04	5.50E-05	9.886	0.338
**XRCC3**	cg23861457	14	104173299	-0.149	1.45E-03	1.11E-04	8.115	0.856
**FANCM**	cg19772317	14	45605172	-0.130	5.71E-03	4.02E-04	10.208	0.049
**MRE11A**	cg07258852	11	94227583	-0.111	1.84E-02	1.21E-03	8.713	0.127
**CHD1**	cg21082921	5	98263013	-0.110	1.88E-02	1.23E-03	9.615	0.087
**BRIP1**	cg00515161	17	59940900	-0.105	2.60E-02	1.67E-03	6.085	0.030
**TP53**	cg16203911	17	7590731	-0.088	6.12E-02	3.79E-03	10.208	0.054

Supplementary Table 1: *LUAD-specific methylation probes used to determine the methylation status of the promoters of the HR-related genes. The Meth_Mean column contains the mean beta value of the selected methylation probe.*

Gene	Meth_Probe	Chrom	Position	Corr_Coeff	Pval	Qval	Expr_Mean	Meth_Mean
**PIK3C2G**	cg17881542	12	18415070	-0.5184	0	0	3.2302152	0.568849
**FANCC**	cg13633659	9	98012426	-0.4887	0	0	8.2236032	0.587572
**XRCC3**	cg23193616	14	104177530	-0.4836	0	0	8.9659951	0.416457
**FANCG**	cg05293216	9	35080812	-0.4253	0	0	8.9808336	0.614884
**RAD51C**	cg19208681	17	56769628	-0.393	3.089E-15	1.011E-15	8.2994915	0.526623
**ERCC2**	cg01587190	19	45874071	-0.3418	1.88E-11	3.895E-12	8.8712109	0.058187
**DIS3**	cg14756530	13	73355442	-0.3125	9.833E-10	1.618E-10	8.3182011	0.036279
**PARP3**	cg27227100	3	51977385	-0.3086	1.61E-09	2.569E-10	8.2097129	0.853407
**BRCA1**	cg25067162	17	41277974	-0.3031	3.204E-09	4.911E-10	8.9337162	0.23001
**BRIP1**	cg05377417	17	59940892	-0.273	1.072E-07	1.341E-08	7.4324743	0.028383
**FANCM**	cg03491181	14	45605002	-0.2268	1.11E-05	1.071E-06	10.1984903	0.039346
**MRE11A**	cg08831996	11	94227006	-0.2228	1.594E-05	1.511E-06	8.9838036	0.021479
**ATM**	cg14761454	11	108092087	-0.2019	0.0001061	9.214E-06	9.6776746	0.199893
**XRCC2**	cg05898482	7	152373203	-0.1592	0.002154	0.0001646	7.0688719	0.058785
**FANCM**	cg16941144	14	45603841	-0.1545	0.00291	0.0002198	7.168296	0.034481
**PALB2**	cg04035070	16	23653174	-0.134	0.009913	0.0007127	8.736369	0.023335
**CHD1**	cg21082921	5	98263013	-0.07359	0.1577	0.0102	9.3214453	0.058536
**BRCA2**	cg15865175	13	32890215	-0.06146	0.2382	0.01513	7.4723209	0.031392

Supplementary Table 2: *LUSC-specific methylation probes used to determine the methylation status of the promoters of the HR-related genes. The Meth_Mean column contains the mean beta value of the selected methylation probe.*

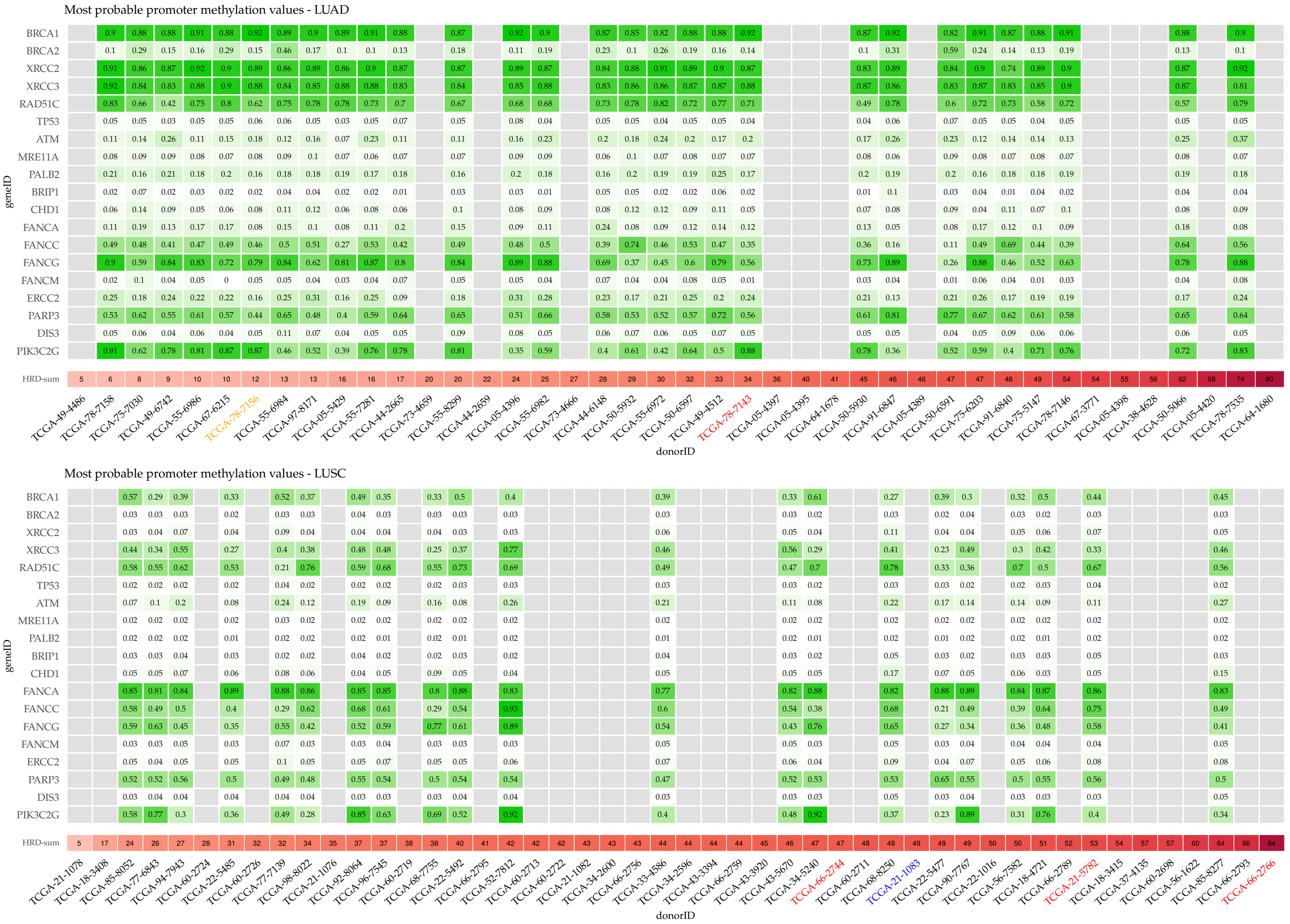

Supplementary Figure 4: *Array-based (Human Methylation 450k) promoter methylation states of HR-related genes in the tumors of the TCGA lung adenocarcinoma (above) and squamous cell carcinoma (below) cohorts. The tiles of the top panels are colored according to the beta-values of the selected probes, which are also indicated as rounded numbers in each tile. Samples are ordered according to the sum of their genomic scar scores (HRD-LOH, HRD-LST, HRD-TAI). Samples with grey tiles had no available 450k methylation data.*

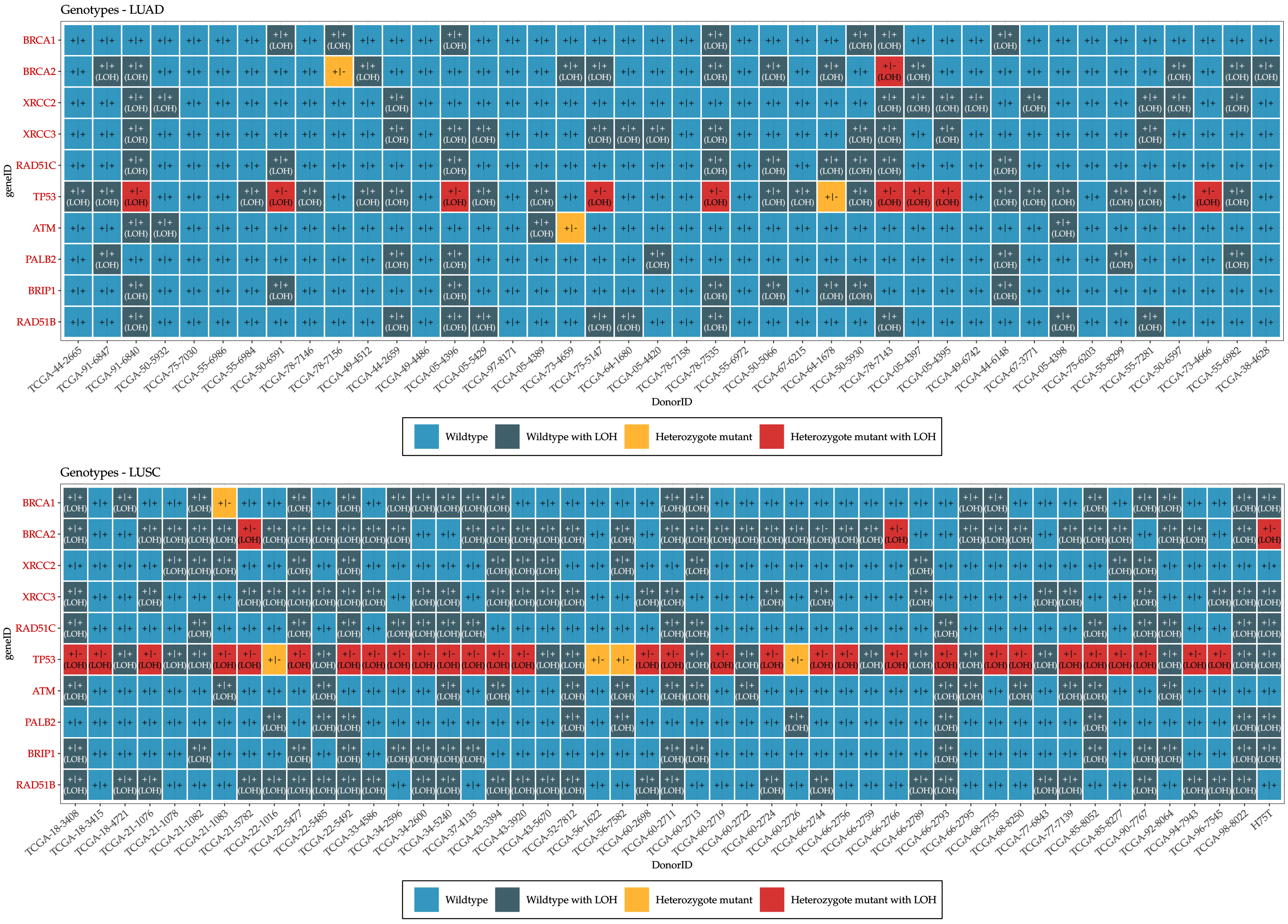

Supplementary Figure 5: *Final genotypes of the LUAD and LUSC WGS cohorts. Genotyping is based on the presence of a pathogenic or likely pathogenic somatic/germline mutation in the gene and whether a loss of heterozygosity event accompanies them. - Heterozygote: at least a germline/somatic mutation present, but no LOH, homozygote: at least a germline/somatic mutation present AND an LOH. (Since the germline mutation of TCGA-66-2744 is not present in the tumor, only the LOH is registered.*

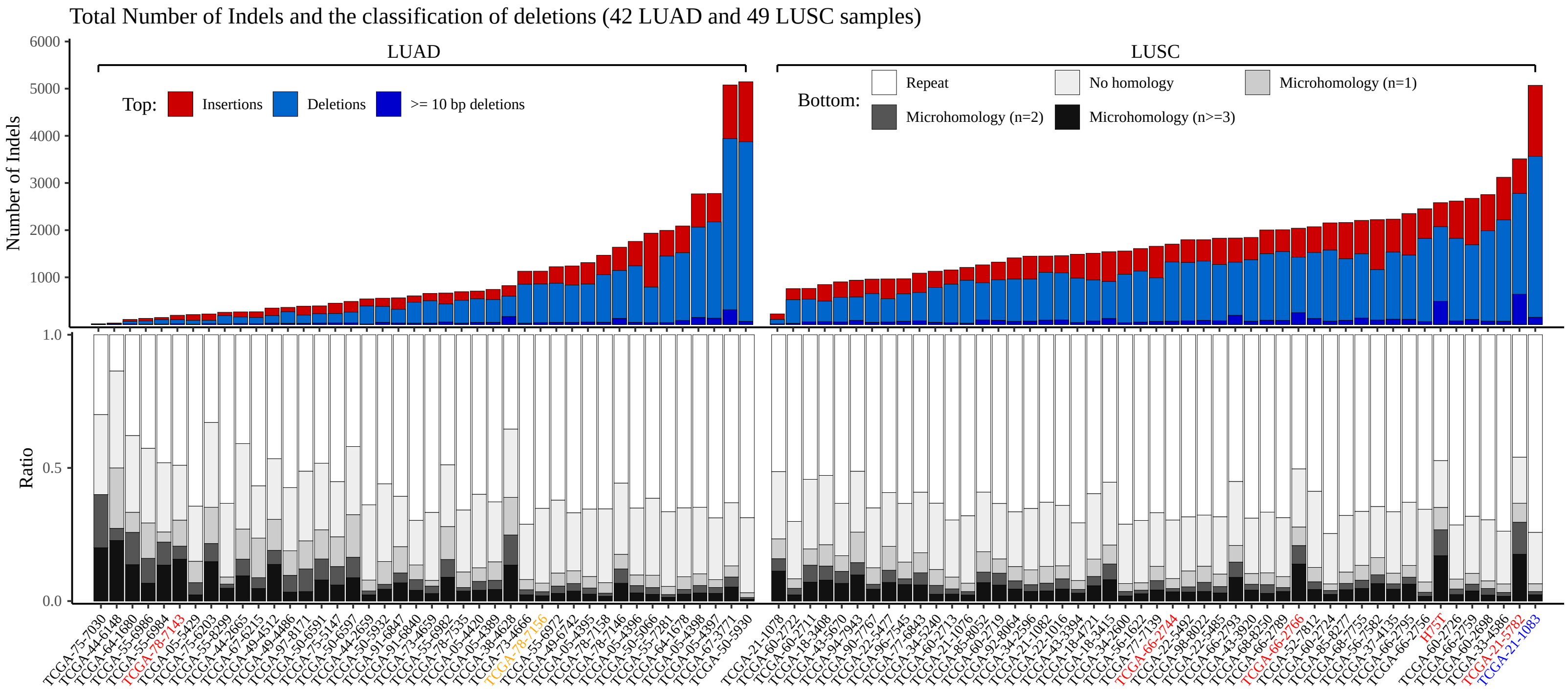

Supplementary Figure 6: *Top panel: Total number of indels in the LUAD and LUSC whole genomes. The horizontal axis contains the absolute number of hard-filtration-passing indels, the bars are colored according to their insertion/deletion content. Deletions are futher divided into <10 and ≥ 10 bp deletions.*

Bottom panel: Relative constituents of deletions according to the deletion-classification scheme. The three major categories are repeats, microhomologies and deletions without homologous recurrences. Microhomologies are further divided into n=1 bp, n=2 bp, and n≥3 bp variants.

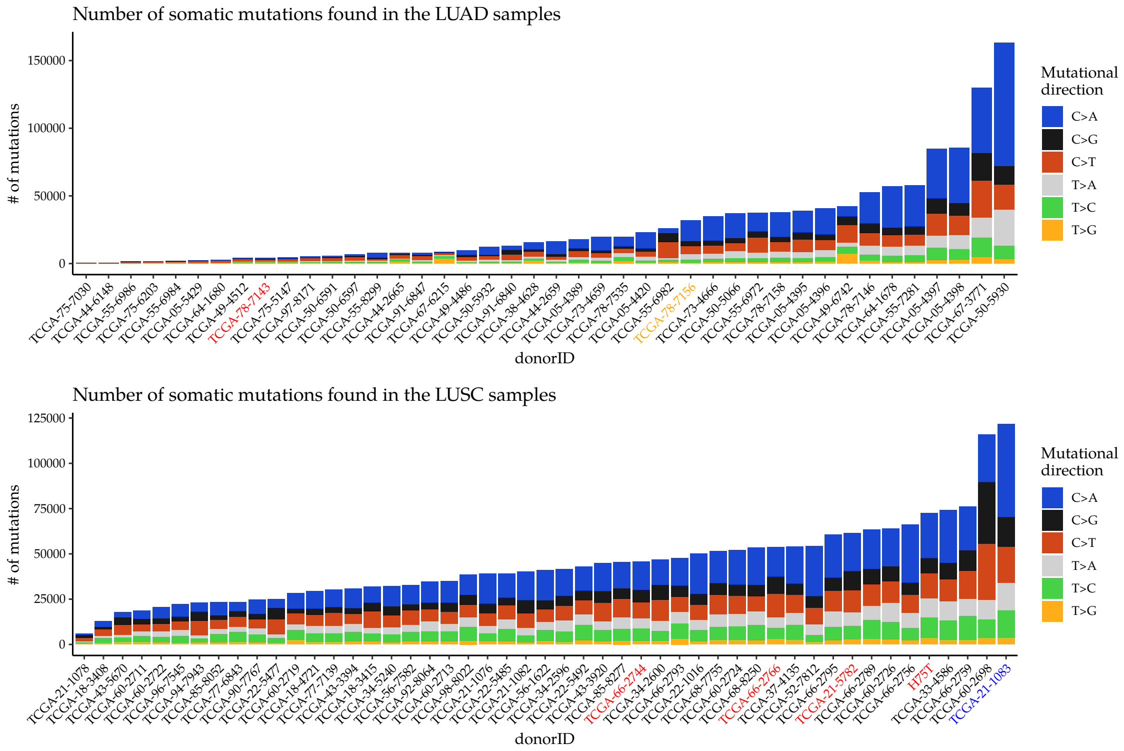

Supplementary Figure 7: *Summary of the somatic substitutions detected in the whole genome cohorts. The vertical axis contains overall numbers, the colors on the bars indicate the relative composition of the mutational directions. Most samples are dominated by C>A mutations. Top panel: LUAD, Bottom panel: LUSC.*

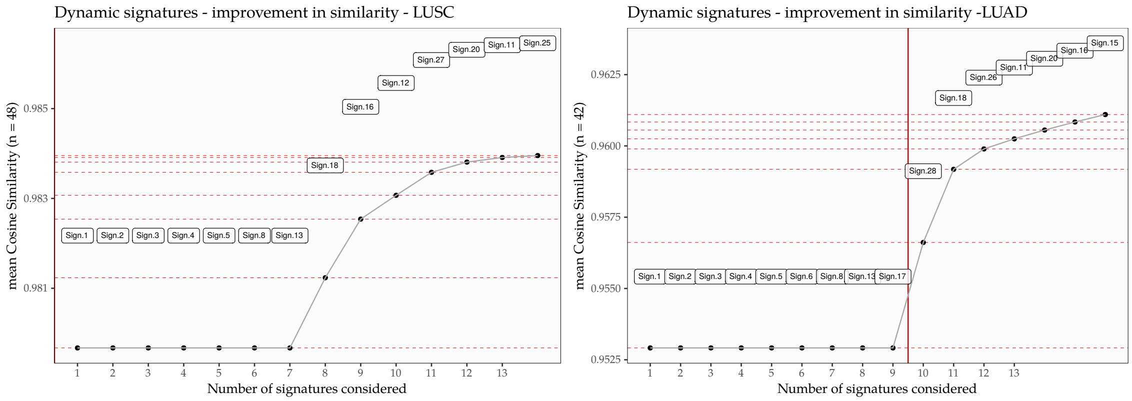

Supplementary Figure 8: *A graphical representation of the dynamic signature extraction process. In each iteration a signature that would improve the most on the cosine similarities were considered, however no additional signatures were added to the list in either cohort. Left: LUSC, Right: LUAD*

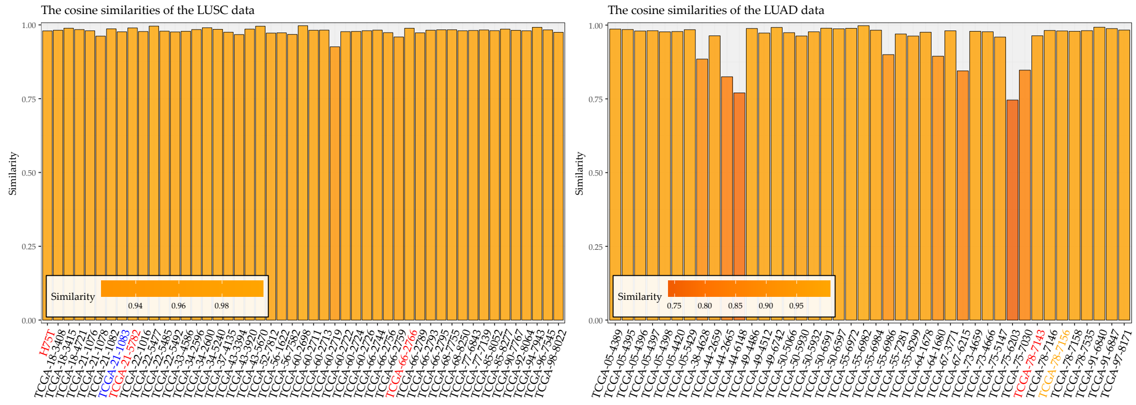

Supplementary Figure 9: *Cosine similarities between the original and reconstructed mutational alphabets. Left: LUSC, Right: LUAD*

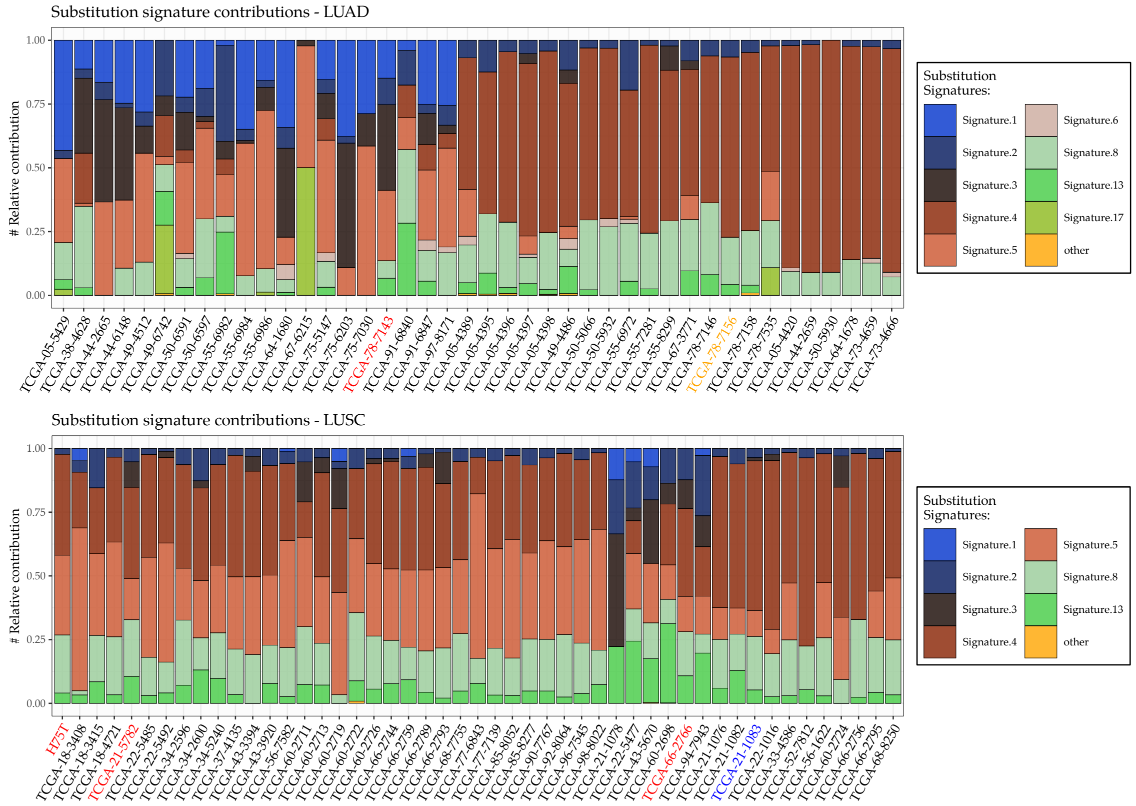

Supplementary Figure 10: *Somatic signature composition of the LUAD and LUSC whole genomes. Top panel: LUAD, Bottom panel: LUSC.*

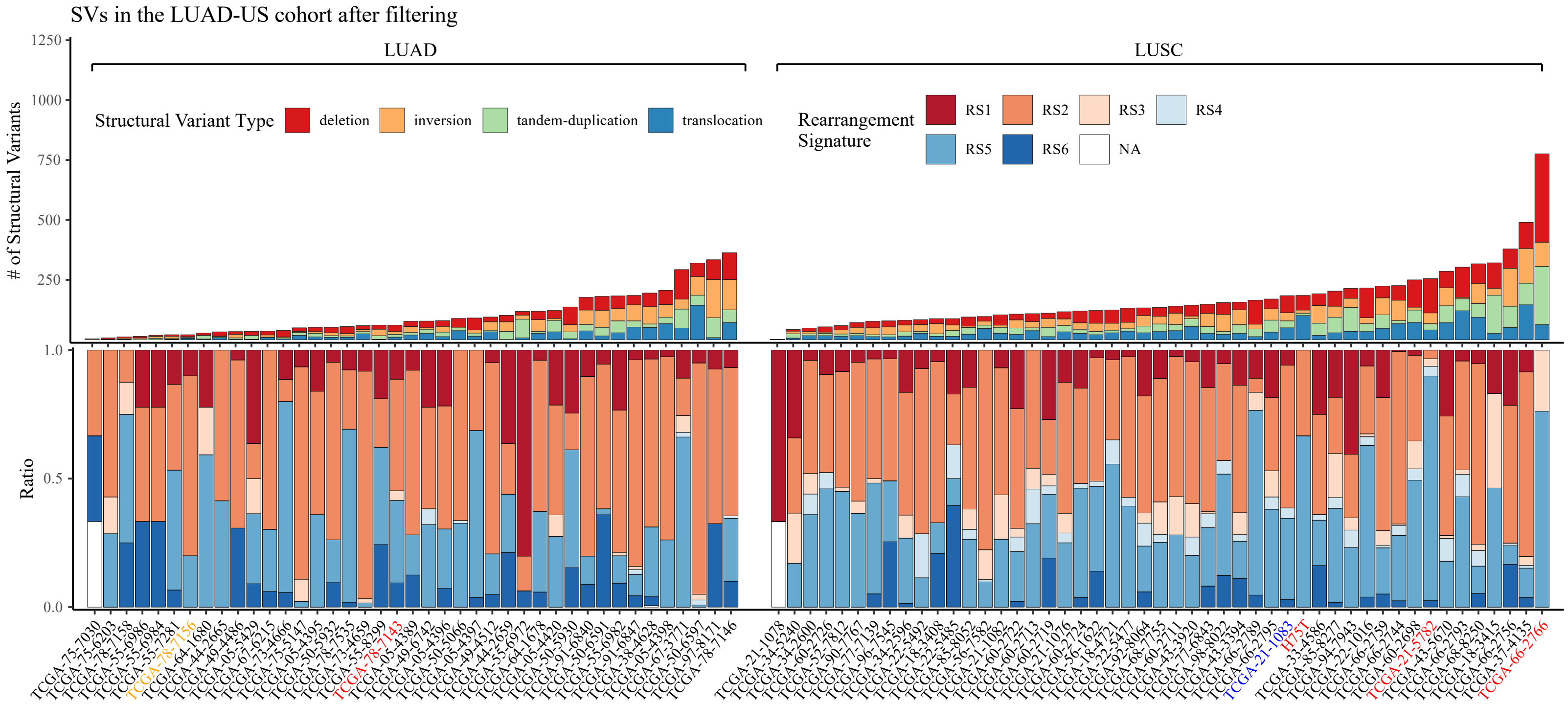

Supplementary Figure 11: *Top panel: Hard-filter-passing structural variants present in the LUAD and LUSC WGS cohorts. The vertical axis shows the total number of structural variants in each sample, on the horizontal axis samples are sorted according to this number.*

Bottom panel: Rearrangement signatures in the two lung cancer cohorts. The bars only show the relative compositions, the order of the samples follows the order of the structural variant (top) plot.

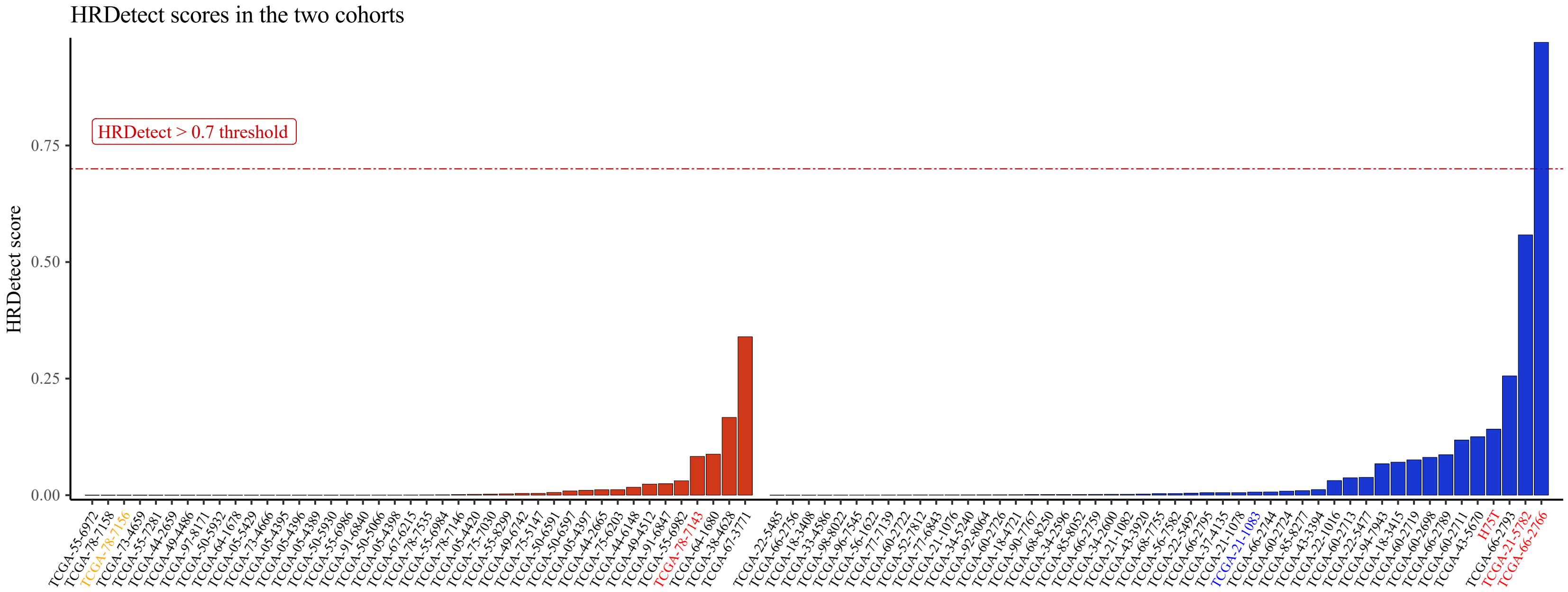

Supplementary Figure 12: *Breast cancer standardized HRDetect scores of the LUAD and LUSC whole genomes.*

tcga_donor_id	TCGA-21-1083	TCGA-21-5782	TCGA-66-2744	TCGA-66-2766	H75T
**cohort**	LUSC	LUSC	LUSC	LUSC	LUSC
**gender**	male	female	male	male	male
**prior malignancy**	Yes, undefined prior	none	none	none	none
**therapy**	Not available	Not available	Not available	Not available	gemcitabine-carboplatin
**response to therapy**	Not available	Not available	Not available	Not available	Partial Response
**genotype**	BRCA1 het. mutant	BRCA2 hom. mutant	BRCA2 hom. mutant	BRCA2 hom. mutant	BRCA2 hom. mutant
**donor_age_at_diagnosis**	75	68	71	54	76
**donor_age_at_last_followup**	75	70	71	54	79
**vital_status**	deceased	deceased	alive	alive	alive
**disease_status_last_followup**	-	-	complete remission	complete remission	complete remission
**numberOfIndels**	5071	3513	1704	2042	2584
**numberOfInsertions**	1506	734	379	615	511
**numberOfDeletions**	3565	2779	1325	1427	2073
**delInsRatio**	2.367	3.786	3.496	2.320	4.057
**numberOf(>9bp)Deletions**	153	641	71	250	493
**fractionOf(>9bp)Deletions**	0.043	0.231	0.054	0.175	0.238
**RS1**	10	0	1	0	0
**RS2**	95	8	135	0	23
**RS3**	0	7	1	187	0
**RS4**	7	9	8	0	0
**RS5**	54	208	51	600	46
**RS6**	5	6	5	0	0
**RS1_ratio**	0.058	0.000	0.005	0.000	0.000
**RS2_ratio**	0.556	0.034	0.672	0.000	0.333
**RS3_ratio**	0.000	0.029	0.005	0.238	0.000
**RS4_ratio**	0.041	0.038	0.040	0.000	0.000
**RS5_ratio**	0.316	0.874	0.254	0.762	0.667
**RS6_ratio**	0.029	0.025	0.025	0.000	0.000
**HRD_LOH**	17	20	17	26	11
**HRD_LST**	15	16	8	29	35
**HRD_TAI**	17	17	22	29	27
**numberOfMicrohom**	232	1021	112	397	729
**numberOfMicrohom(>2bp)**	84	489	46	198	353
**numberOfRepeats**	2646	1278	922	719	980
**numberOfUniques**	687	480	291	311	364
**Signature.1**	0	0	0	0	0
**Signature.2**	143	400	249	2017	889
**Signature.3**	739	179	140	2151	1252
**Signature.4**	14669	3459	20389	12631	22000
**Signature.5**	34	717	668	11369	0
**Signature.8**	783	8124	3866	308	8318
**Signature.13**	927	4105	4250	1588	0
**Signature.1_ratio**	0.000	0.000	0.000	0.000	0.000
**Signature.2_ratio**	0.036	0.052	0.039	0.123	0.027
**Signature.3_ratio**	0.013	0.100	0.011	0.113	0.039
**Signature.4_ratio**	0.587	0.358	0.422	0.345	0.678
**Signature.5_ratio**	0.102	0.162	0.280	0.138	0.000
**Signature.8_ratio**	0.210	0.222	0.170	0.174	0.256
**Signature.13_ratio**	0.053	0.106	0.077	0.108	0.000

Supplementary Table 3: *Genomic features and clinical data of lung squamous carcinoma samples with mutations in key HR genes.*

tcga_donor_id	TCGA-78-7143	TCGA-78-7156	TCGA-64-1680
**cohort**	LUAD	LUAD	LUAD
**gender**	female	male	male
**prior malignancy**	none	none	wild-type
**therapy**	Not available	Not available	Not available
**response to therapy**	Not available	Not available	Not available
**genotype**	BRCA2 hom. mutant	BRCA2 get. mutant	RAD51B hom. mutant
**donor_age_at_diagnosis**	62	62	63
**donor_age_at_last_followup**	62	62	66
**vital_status**	deceased	deceased	alive
**disease_status_last_followup**	-	-	complete remission
**numberOfIndels**	196	1131	107
**numberOfInsertions**	94	272	41
**numberOfDeletions**	102	859	66
**delInsRatio**	1.091	3.162	1.641026
**numberOf(>9bp)Deletions**	17	40	14
**fractionOf(>9bp)Deletions**	0.167	0.047	0.21212121
**RS1**	6	2	6
**RS2**	23	14	0
**RS3**	2	0	5
**RS4**	0	0	0
**RS5**	17	4	16
**RS6**	5	0	0
**RS1_ratio**	0.113	0.100	0.222222222
**RS2_ratio**	0.434	0.700	0
**RS3_ratio**	0.038	0.000	0.185185185
**RS4_ratio**	0.000	0.000	0
**RS5_ratio**	0.321	0.200	0.5925926
**RS6_ratio**	0.094	0.000	0
**HRD_LOH**	5	5	7
**HRD_LST**	2	1	47
**HRD_TAI**	27	6	26
**numberOfMicrohom**	31	58	22
**numberOfMicrohom(>2bp)**	16	17	9
**numberOfRepeats**	50	560	25
**numberOfUniques**	21	241	19
**Signature.1**	589	0	895
**Signature.2**	412	2080	214
**Signature.3**	1332	0	913
**Signature.4**	0	22454	0
**Signature.5**	1094	0	282
**Signature.8**	273	5888	132
**Signature.13**	266	1349	30
**Signature.1_ratio**	0.149	0.000	0.3415248
**Signature.2_ratio**	0.104	0.065	0.08169609
**Signature.3_ratio**	0.336	0.000	0.34851058
**Signature.4_ratio**	0.000	0.707	0
**Signature.5_ratio**	0.276	0.000	0.107461
**Signature.8_ratio**	0.069	0.185	0.05042454
**Signature.13_ratio**	0.067	0.042	0.01134194

Supplementary Table 4: *Genomic features and clinical data of lung adenocarcinoma samples with mutations in key HR genes.*

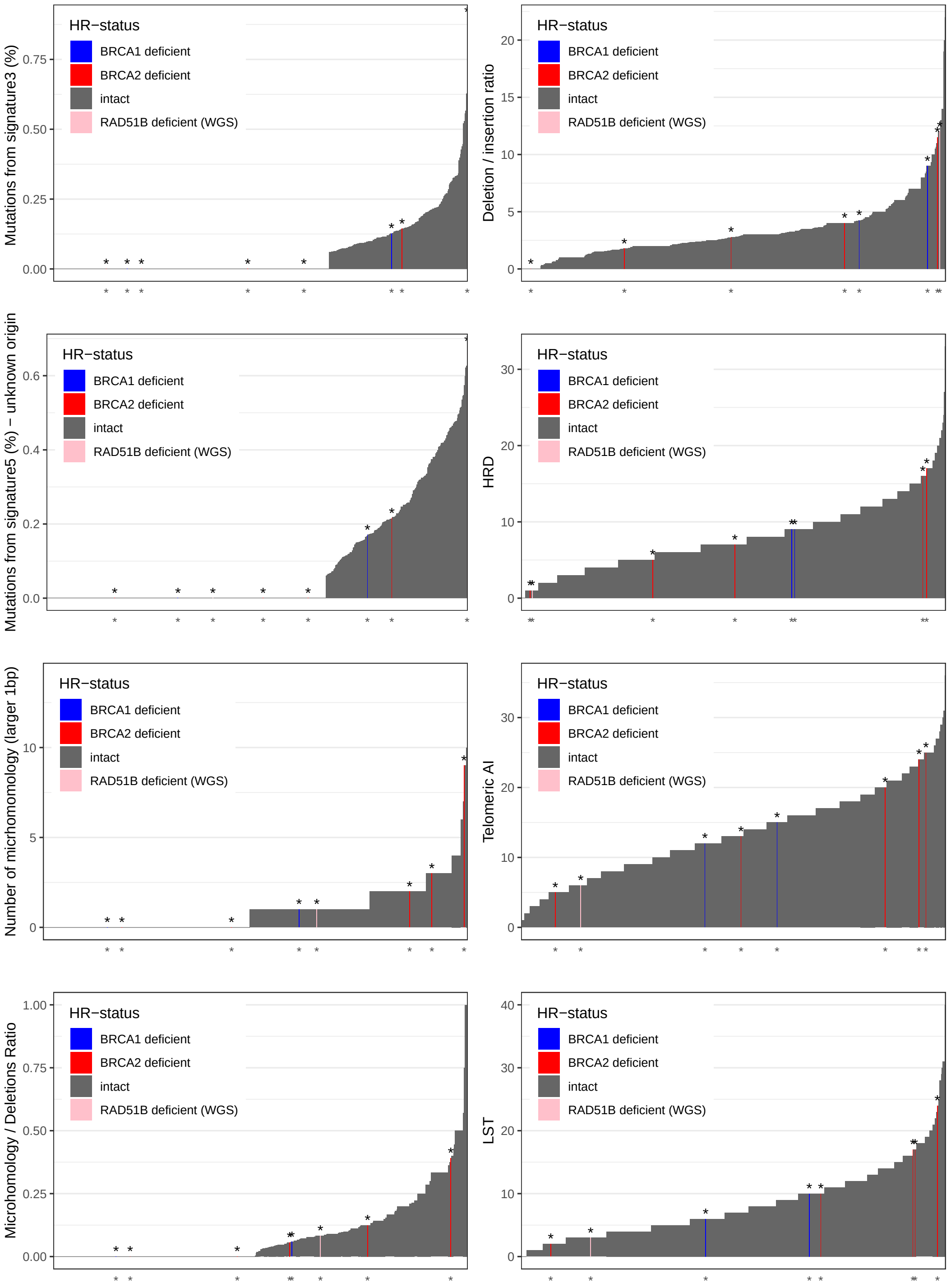

Supplementary Figure 13: *Distribution of genomic scar scores (HRD-LOH, Telomeric Allelic Imbalance, Large-scale Transitions), homologous-recombination deficiency related mutational signatures (Signature 3, 5), number of micrhomomology-mediated deletions, microhomology / deletions ratio, deletion / insertion ratio and BRCA1/2-status in whole exome sequenced lung adenocarcinoma samples (LUAD, n=553).*

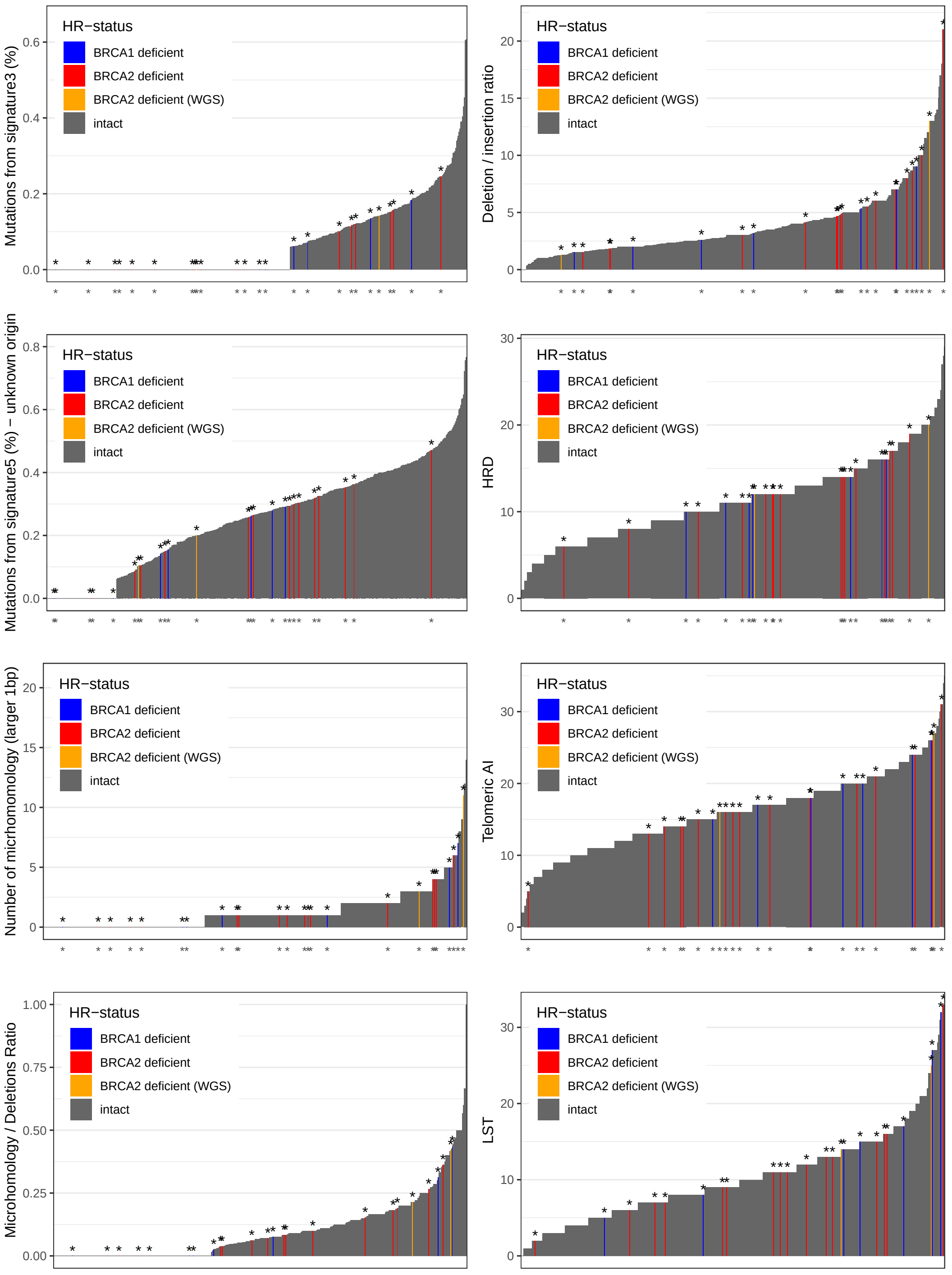

Supplementary Figure 14: *Distribution of genomic scar scores (HRD-LOH, Telomeric Allelic Imbalance, Large-scale Transitions), homologous-recombination deficiency related mutational signatures (Signature 3, 5), number of micrhomomology-mediated deletions, microhomology / deletions ratio, deletion / insertion ratio and BRCA1/2-status in whole exome sequenced lung squamous carcinoma samples (LUSC, n=489).*

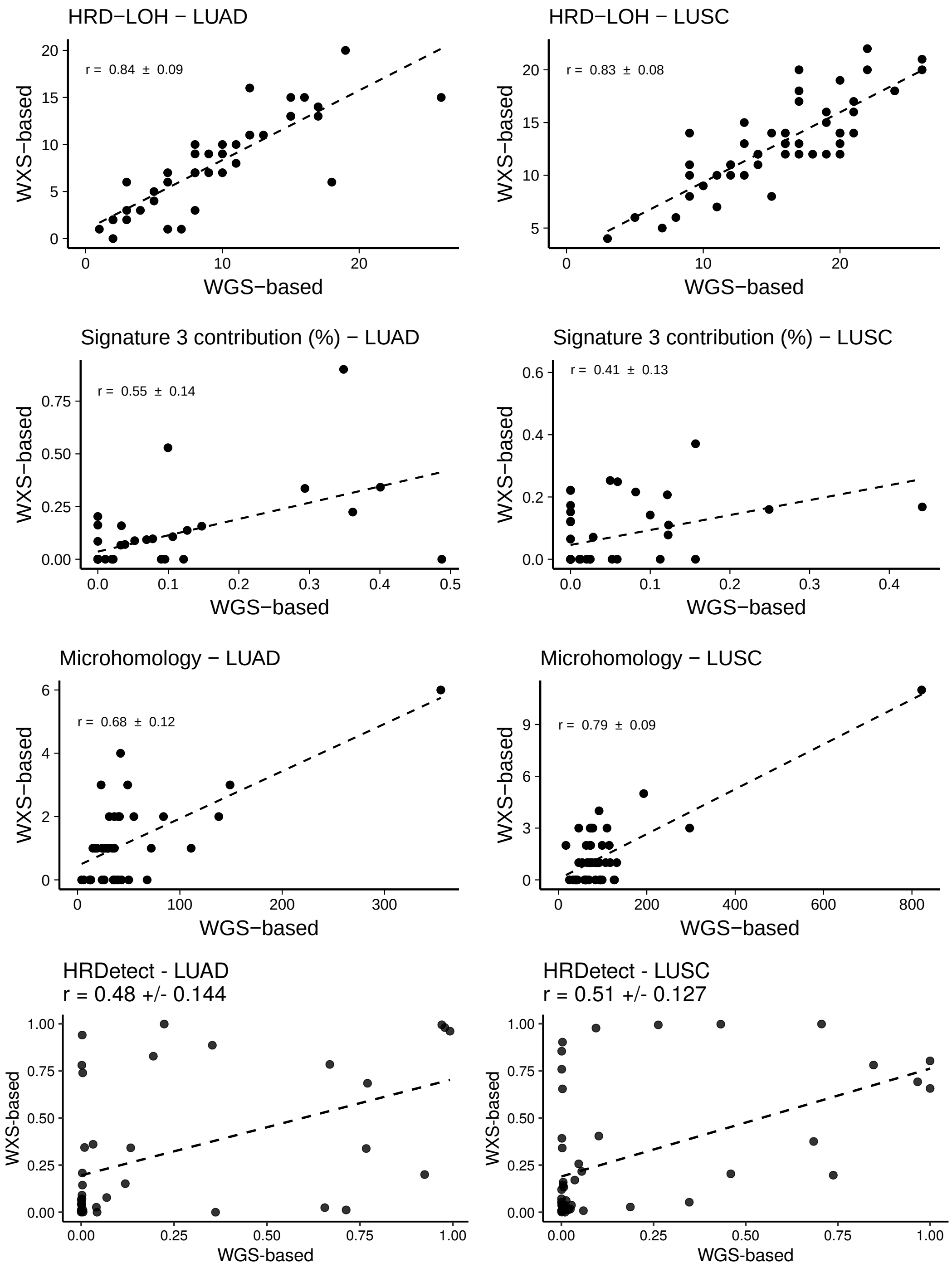

Supplementary Figure 15: *Correlation of the three main components (number of HRD-LOH events, number of microhomology-mediated deletions, and contribution of Signature 3 to the mutational profile) of HRDetect between paired whole exome and whole genome sequenced samples*

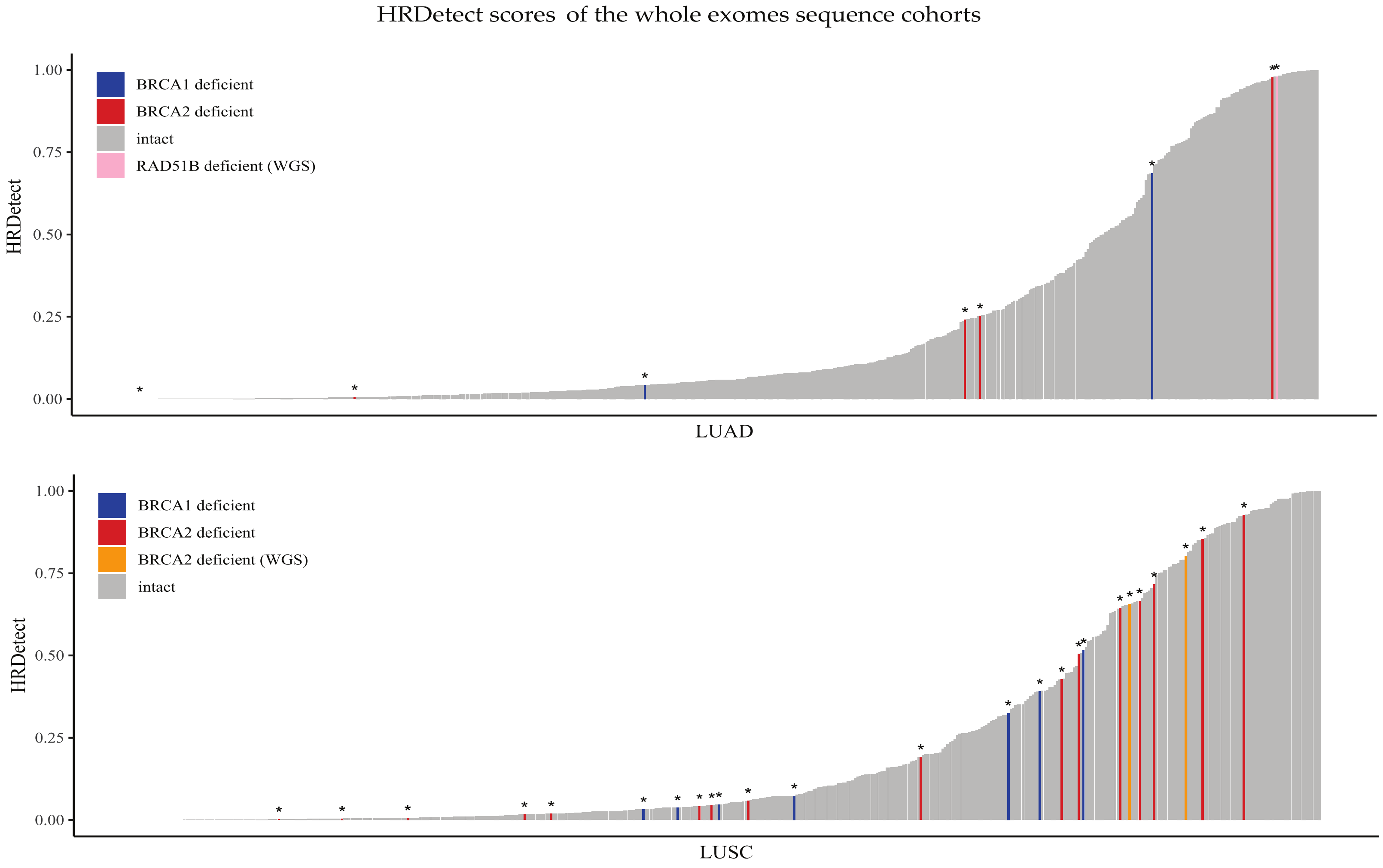

Supplementary Figure 16: *Breast cancer standardized HRDetect scores of the LUAD and LUSC whole exome (TCGA).*

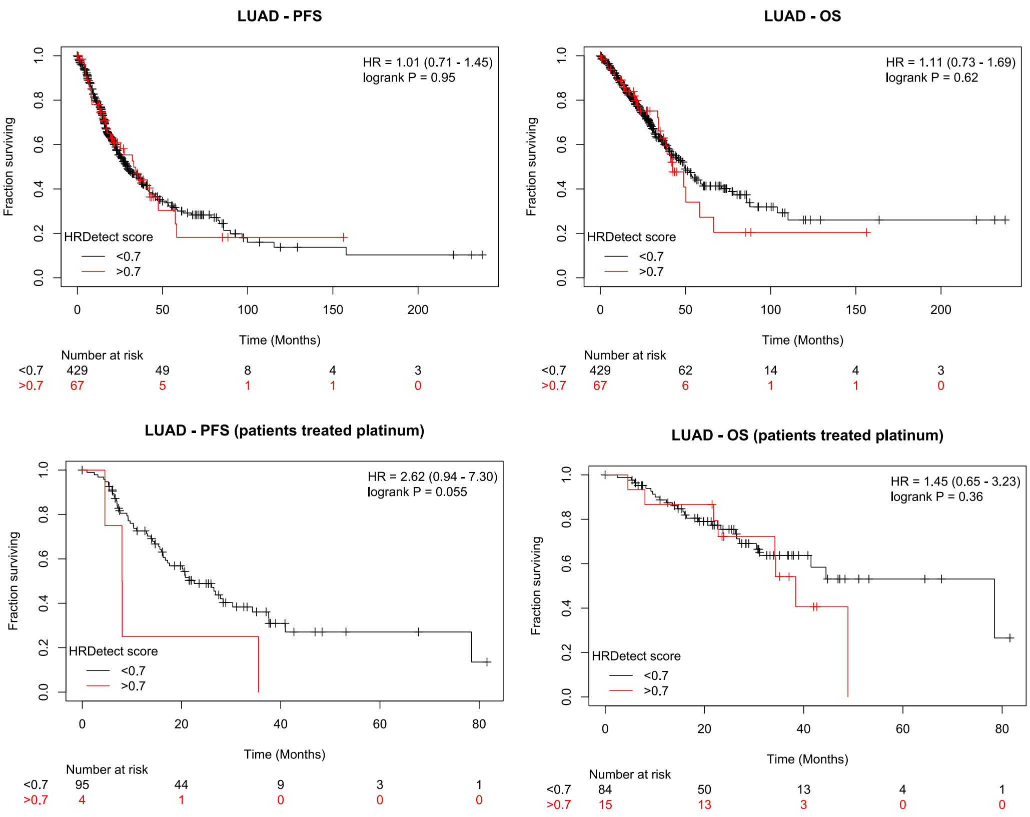

Supplementary Figure 17: *Survival curves - LUAD*

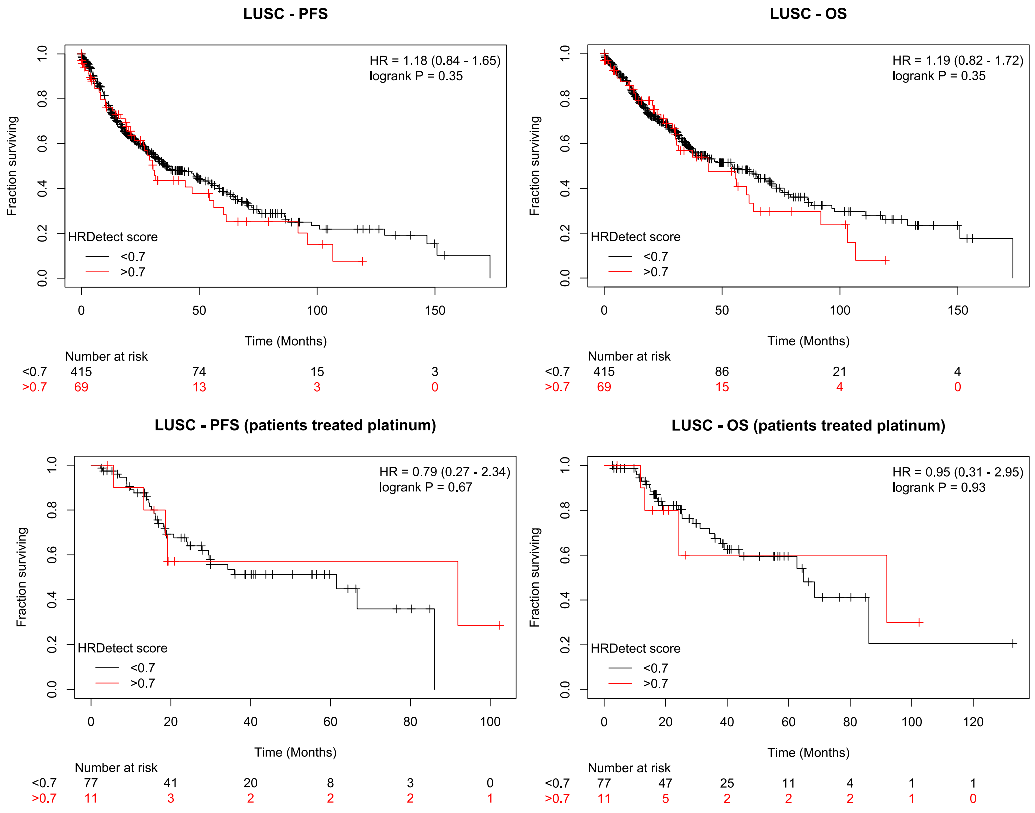

Supplementary Figure 18: *Survival curves - LUSC*

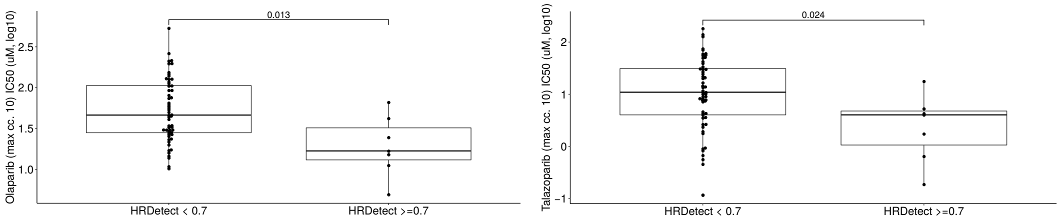

Supplementary Figure 19: *Lung cancer cell lines with larger than 0.70 HRDetect scores showed significantly (p<0.05) higher sensitivity to olaparib and talazoparib based on CCLE and GDSC data. x-axis: fitted* $\log_{10}$ *IC50(μM). The p-values are from Wilcoxon rank sum tests.*

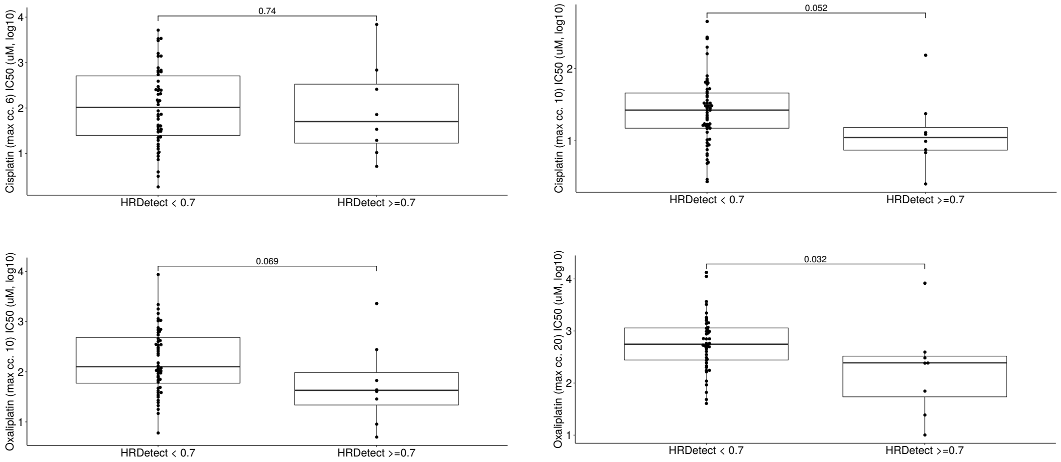

Supplementary Figure 20: *Lung cancer cell lines with larger than 0.70 HRDetect scores showed higher sensitivity to platinum-based chemotherapeutic agents cisplatin and oxaliplatin based on and GDSC data. x-axis: Fitted* $\log_{10}$ *IC50(μM). The p-values are from Wilcoxon rank sum tests.*
