# Supplementary material for: A subset of lung cancer cases shows robust signs of homologous recombination deficiency associated genomic mutational signatures
Source: NPJ Precis Oncol. 2021 Jun 18;5:55. doi: 10.1038/s41698-021-00199-8 (PMC8213828; doi:10.1038/s41698-021-00199-8)
Supplement: Supplementary file 4 — Reporting Summary [file 41698_2021_199_MOESM4_ESM.pdf]

nature research

Corresponding author(s): Zoltan Szallasi

Last updated by author(s): Mar 28, 2021

Reporting Summary

Nature Research wishes to improve the reproducibility of the work that we publish. This form provides structure for consistency and transparency in reporting. For further information on Nature Research policies, see our Editorial Policies and the Editorial Policy Checklist.

Statistics

For all statistical analyses, confirm that the following items are present in the figure legend, table legend, main text, or Methods section.

n/a	Confirmed	
☐	☒	The exact sample size (*n*) for each experimental group/condition, given as a discrete number and unit of measurement
☐	☒	A statement on whether measurements were taken from distinct samples or whether the same sample was measured repeatedly
☐	☒	The statistical test(s) used AND whether they are one- or two-sided<br>*Only common tests should be described solely by name; describe more complex techniques in the Methods section.*
☐	☒	A description of all covariates tested
☐	☒	A description of any assumptions or corrections, such as tests of normality and adjustment for multiple comparisons
☐	☒	A full description of the statistical parameters including central tendency (e.g. means) or other basic estimates (e.g. regression coefficient) AND variation (e.g. standard deviation) or associated estimates of uncertainty (e.g. confidence intervals)
☐	☒	For null hypothesis testing, the test statistic (e.g. *F*, *t*, *r*) with confidence intervals, effect sizes, degrees of freedom and *P* value noted<br>*Give P values as exact values whenever suitable.*
☐	☒	For Bayesian analysis, information on the choice of priors and Markov chain Monte Carlo settings
☐	☒	For hierarchical and complex designs, identification of the appropriate level for tests and full reporting of outcomes
☐	☒	Estimates of effect sizes (e.g. Cohen's *d*, Pearson's *r*), indicating how they were calculated

Our web collection on statistics for biologists contains articles on many of the points above.

Software and code

Policy information about availability of computer code

Data collection	TCGA data were downloaded using gdc-client v1.6.0 and icgc-client v0.3.
Data analysis	All analysis was done using standard R (v3.6) codes with the following additional packages: ggplot2, data.table, deconstructSigs, lsa, ggbeeswarm, RColorBrewer, sequenza, copynumber, and cluster. In particular, standard variant files were converted to tab-delimited tables using GATK (v3.8) VariantsToTable and manipulated using the data.table package in R. Figures were created using ggplot2. Every tool mentioned in the Methods section were used with default parameters unless stated otherwise.

For manuscripts utilizing custom algorithms or software that are central to the research but not yet described in published literature, software must be made available to editors and reviewers. We strongly encourage code deposition in a community repository (e.g. GitHub). See the Nature Research guidelines for submitting code & software for further information.

Data

Policy information about availability of data

All manuscripts must include a data availability statement. This statement should provide the following information, where applicable:

- Accession codes, unique identifiers, or web links for publicly available datasets
- A list of figures that have associated raw data
- A description of any restrictions on data availability

The results shown here are in part based upon data generated by the TCGA Research Network: https://www.cancer.gov/tcga, and the LUAD and LUSC data are available at ICGC (https://dcc.icgc.org/) and GDC (https://portal.gdc.cancer.gov/) data portals.
Sample H75T is available upon request from the corresponding author.

Field-specific reporting

Please select the one below that is the best fit for your research. If you are not sure, read the appropriate sections before making your selection.

☒ Life sciences ☐ Behavioural & social sciences ☐ Ecological, evolutionary & environmental sciences

For a reference copy of the document with all sections, see nature.com/documents/nr-reporting-summary-flat.pdf

Life sciences study design

All studies must disclose on these points even when the disclosure is negative.

Sample size	All available samples from the TCGA were chosen for analysis in this study.
Data exclusions	No data were excluded.
Replication	All attempts at replication of the analyses in this study were successful.
Randomization	This is not relevant in this study, as it was based on data analyses rather than experiments.
Blinding	This is not relevant in this study, as it was based on data analyses rather than experiments.

Reporting for specific materials, systems and methods

We require information from authors about some types of materials, experimental systems and methods used in many studies. Here, indicate whether each material, system or method listed is relevant to your study. If you are not sure if a list item applies to your research, read the appropriate section before selecting a response.

Materials & experimental systems

n/a	Involved in the study	
☒	☐	Antibodies
☐	☒	Eukaryotic cell lines
☒	☐	Palaeontology and archaeology
☒	☐	Animals and other organisms
☐	☒	Human research participants
☒	☐	Clinical data
☒	☐	Dual use research of concern

Methods

n/a	Involved in the study	
☒	☐	ChIP-seq
☒	☐	Flow cytometry
☒	☐	MRI-based neuroimaging

Eukaryotic cell lines

Policy information about cell lines

Cell line source(s)	Cancer Cell Line Encyclopedia, Genomics of Drug Sensitivity in Cancer data portal.
Authentication	This is not relevant, all processed data of cell lines were downloaded from their respective sources. No cells were cultured by us.
Mycoplasma contamination	This is not relevant, all processed data of cell lines were downloaded from their respective sources. No cells were cultured by us.
Commonly misidentified lines (See ICLAC register)	No specific cell lines are mentioned in the article, only their collective statistical behavior is studied.

Human research participants

Policy information about studies involving human research participants

Population characteristics	Other than TCGA data, the study involved a single (N=1) exceptional responder.
Recruitment	The exceptional responder was identified as part of a larger ongoing study to understand the determinants of treatment response to platinum based therapy.
Ethics oversight	The Hungarian Scientific and Research Ethics Committee of the Medical Research Council, No 2285-1/2019/EUIG and 2307-3/2020/EUIG has approved the study.

Note that full information on the approval of the study protocol must also be provided in the manuscript.
